# Trio-binning of a hinny refines the comparative organization of the horse and donkey X chromosomes and reveals novel species-specific features

**DOI:** 10.1038/s41598-023-47583-x

**Published:** 2023-11-17

**Authors:** Matthew J. Jevit, Caitlin Castaneda, Nandina Paria, Pranab J. Das, Donald Miller, Douglas F. Antczak, Theodore S. Kalbfleisch, Brian W. Davis, Terje Raudsepp

**Affiliations:** 1 School of Veterinary Medicine, Texas A&M University, College Station, TX 77843 USA; 2 Texas Scottish Rite Hospital for Children, Dallas, TX 75219 USA; 3 ICAR-National Research Centre on Pig, Rani, Guwahati, Assam 781131 India; 4 Baker Institute for Animal Health, Cornell University, Ithaca, NY 14853 USA; 5 Maxwell H. Gluck Equine Research Center, University of Kentucky, Lexington, KY 40546 USA

**Keywords:** Evolution, Molecular evolution

## Abstract

We generated single haplotype assemblies from a hinny hybrid which significantly improved the gapless contiguity for horse and donkey autosomal genomes and the X chromosomes. We added over 15 Mb of missing sequence to both X chromosomes, 60 Mb to donkey autosomes and corrected numerous errors in donkey and some in horse reference genomes. We resolved functionally important X-linked repeats: the *DXZ4* macrosatellite and ampliconic Equine Testis Specific Transcript Y7 (*ETSTY7*). We pinpointed the location of the pseudoautosomal boundaries (PAB) and determined the size of the horse (1.8 Mb) and donkey (1.88 Mb) pseudoautosomal regions (PARs). We discovered distinct differences in horse and donkey PABs: a testis-expressed gene, *XKR3Y*, spans horse PAB with exons1–2 located in Y and exon3 in the X–Y PAR, whereas the donkey *XKR3Y* is Y-specific. *DXZ4* had a similar ~ 8 kb monomer in both species with 10 copies in horse and 20 in donkey. We assigned hundreds of copies of *ETSTY7*, a sequence horizontally transferred from *Parascaris* and massively amplified in equids, to horse and donkey X chromosomes and three autosomes. The findings and products contribute to molecular studies of equid biology and advance research on X-linked conditions, sex chromosome regulation and evolution in equids.

## Introduction

The X chromosome is the most conserved and the best studied mammalian chromosome^1, 2^ owing to the unique features that distinguish it from the autosomes. The chromosome carries disproportionately more genes for muscle and brain functions compared to autosomes^3^, is enriched for hybrid sterility factors such as the *DXZ4* macrosatellite^4^, and may carry testis- and lineage-specific ampliconic gene families with functions in reproduction and sex chromosome meiotic drive^4–8^. Hemizygosity of the X chromosome in males exposes recessive phenotypes and has allowed mapping X-linked disorders long before autosomal gene mapping tools became available^1, 9^. Random inactivation of one of the two X chromosomes (XCI) in mammalian females^10, 11^ buffers the effect of X-linked mutations, so that X chromosome abnormalities in females are more viable than autosomal mutations^12, 13^. On the other hand, knowledge about XCI, has revealed that the genes in the pseudoautosomal region (PAR), gametologs (XY ancestral genes) and some X-specific genes, escape XCI^14–16^. The escape genes are important dosage sensitive regulators for normal development and contribute to sexual dimorphism of X-linked conditions^15, 17, 18^. Despite the biomedical importance and evolutionary interest, the sequence assembly of the X chromosome in most animal reference genomes lags those of the autosomes. The main challenge of studying the X chromosome, is its structural complexity: the X is enriched with interstitial repeats, such as long interspersed nuclear elements L1 (LINE L1) and long terminal repeats (LTRs), has over ten times more segmental duplications compared to the autosomal genome^1^, and carries functionally important repeats such as *DXZ4*^4^ and is replete with large amplicons^5, 7^.

Currently, the most frequently used approach for research and clinical sequencing is the highly accurate and relatively inexpensive short-read Illumina technology^19, 20^ which, however, has limited ability to assemble the most structurally complex parts of the genome, such as segmental duplications, tandem repeats, amplicons, large copy number variants, and inversions^21–26^. These difficulties have been overcome by combining ultra-long Oxford Nanopore reads and PacBio high-fidelity (HiFi) circular consensus long-reads to produce Telomere-to-Telomere (T2T) assembly of the human X chromosome^21^, human chromosome 8^27^ and the complete gapless sequence of the human genome^28^, including the Y chromosome^29^. While this strategy is extremely powerful, it is also prohibitively expensive for most non-human species. An alternative for improved assembly of structurally complex regions is trio-binning which uses short reads from two parental genomes to partition long reads from their offspring into haplotype-specific bins, followed by independent assembly of each haplotype^23^. Trio-binning has been successfully applied to mammalian interspecific F1 hybrids to obtain high-quality haploid assemblies of the two parent species, e.g., cattle and American bison^30^, cattle and yak^31^, and cat and Asian Leopard cat^32^.

Here we carry out trio-binning of a female hinny, the F1 hybrid of a horse stallion and a jenny donkey, to improve the assemblies of the horse and donkey X chromosomes, define and characterize the sequence of the pseudoautosomal boundary, and resolve sequences of functionally important ampliconic arrays and repeats. A significant byproduct of trio-binning is a refined haploid assembly of the horse genome and a significantly improved haploid assembly of the donkey genome.

## Results

### The horse and donkey X chromosomes

**Figure 1:**
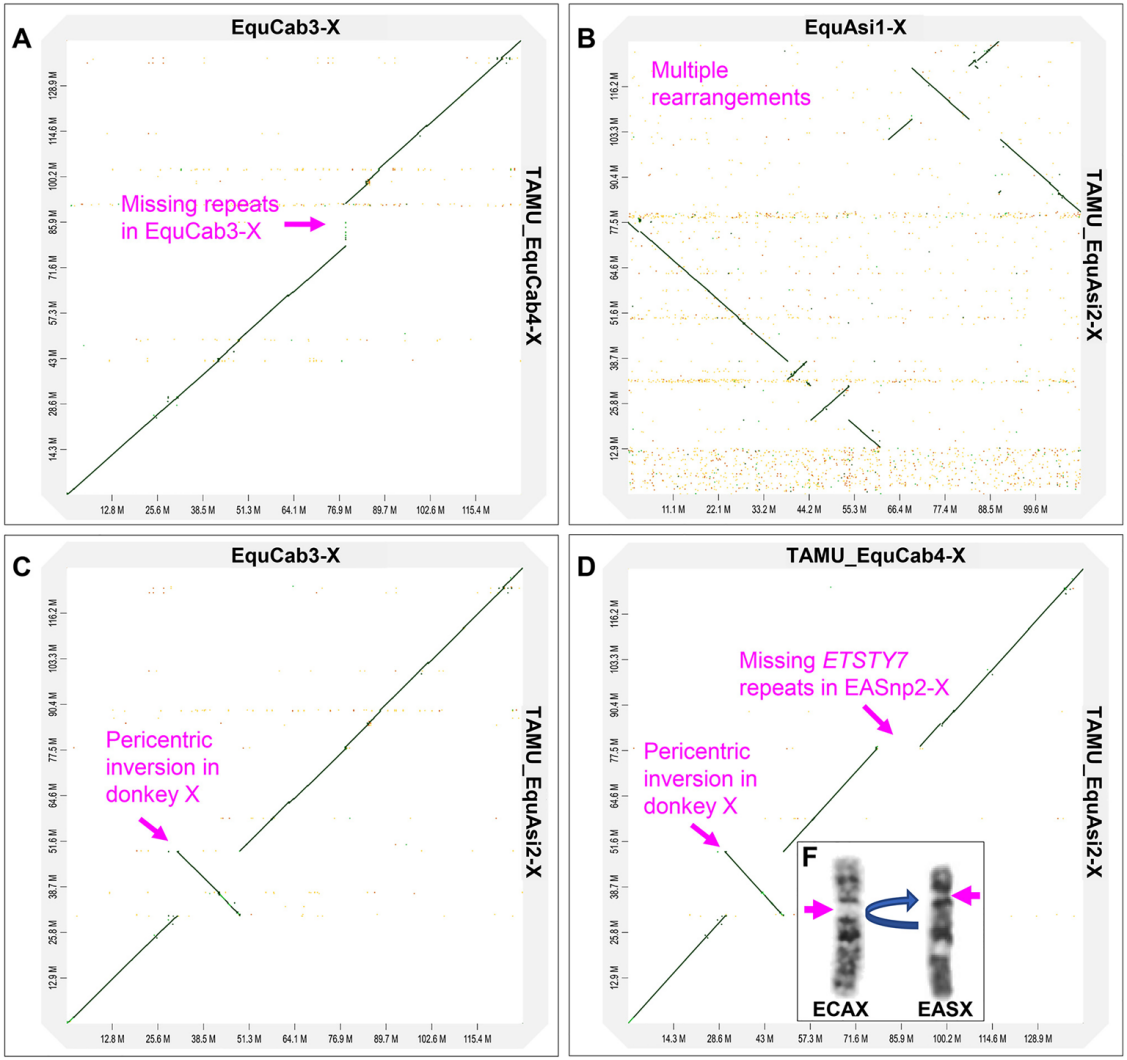
Dot plots alignments using D-genies. (**A**) Newly assembled horse X chromosome (TAMU_EquCab4-X) with horse reference EquCab3-X; (**B**) Newly assembled donkey X chromosome (TAMU_EquAsi2-X) with donkey reference EquAsi1-X; (**C**) TAMU_EquAsi2-X with horse reference EquCab3-X; (**D**) TAMU_EquAsi2-X with TAMU_EquCab4-X; an arrow indicates pericentric inversion in donkey X; (**F**) G-banded horse (ECA) and donkey (EAS) X chromosomes, purple arrows showing centromere positions and a blue arrow indicating pericentric inversion in the donkey X.

The horse and donkey X chromosomes, TAMU_EquCab4-X and TAMU_EquAsi2-X, that were obtained from trio-binned assemblies improved the published horse and donkey reference X chromosomes, EquCab3-X^33^ and EquAsi1-X^34^, in size, contiguity, and accuracy (Table 1). The final horse X chromosome, TAMU_EquCab4-X, was 143,200,399 base pairs (bp), exceeding EquCab3-X by almost 15 mega bases (Mb). Despite the size differences, the two horse X chromosomes aligned nearly perfect (Fig. 1A). Among the primary improvements implemented in TAMU_EquCab4-X, were resolving complex sequences at the pseudoautosomal boundary (PAB) and the incorporation of multiple copies of the *DXZ4* macrosatellite and Equine Testis Specific Transcript Y7 (*ETSTY7*) ampliconic array in the long arm (Xq). In contrast, alignment of stepwise assemblies (see Materials and Methods) of the donkey X chromosome with the donkey X chromosome reference^34^ indicated, even at the contig level, that the published EquAsi1-X is not reliable and could not be used (Fig. 1B). Instead, the donkey X chromosome was assembled using EquCab3-X and the newly assembled TAMU_EquCab4-X (Fig. 1C,D). The new TAMU_EquAsi2-X was over 18 Mb larger than EquAsi1-X (Table 1) and corrected numerous assembly errors (Table S7) in the reference^34^. Alignment of final horse and donkey X chromosomes with each other showed extensive collinearity but also indicated for the first time at sequence-level, the location of a cytogenetically known^35^ pericentric inversion in TAMU_EquAsi2 (Fig. 1D,F). The inversion breakpoints were assigned to ~ 31 Mb and ~ 49 Mb in the horse X chromosomes, EquCab3-X and TAMU_EquCab4-X, and the size of the inverted segment in the donkey X chromosome was approximately 18 Mb (Fig. S2).

**Table 1:** Assembly metrics of the horse and donkey X chromosomes and annotation for PAR-PAB, *DXZ4*, and *ETSTY7.*

Species	References	Size, bp	This study	Size, bp	No. of scaffolds	Scaffold IDs	Scaffold size, bp	Annotation
Horse	EquCab3-X^33^	128,206,784	TAMU_EquCab4-X	143,200,399	5	3351	48,660,579	PAR-PAB
						100032	33,989,383	*ETSTY7*
						100051	9,165,336	*ETSTY7*
						100053	6,738,259	*ETSTY7*
						100026	44,642,821	*DXZ4*
Donkey	EquAsi1-X^34^	110,640,000	TAMU_EquAsi2-X	129,101,567	3	26	30,682,545	PAR-PAB
						100020	47,977,471	*ETSTY7*
						124	50,442,963	*DXZ4*

### The horse pseudoautosomal boundary (PAB)

**Figure 2:**
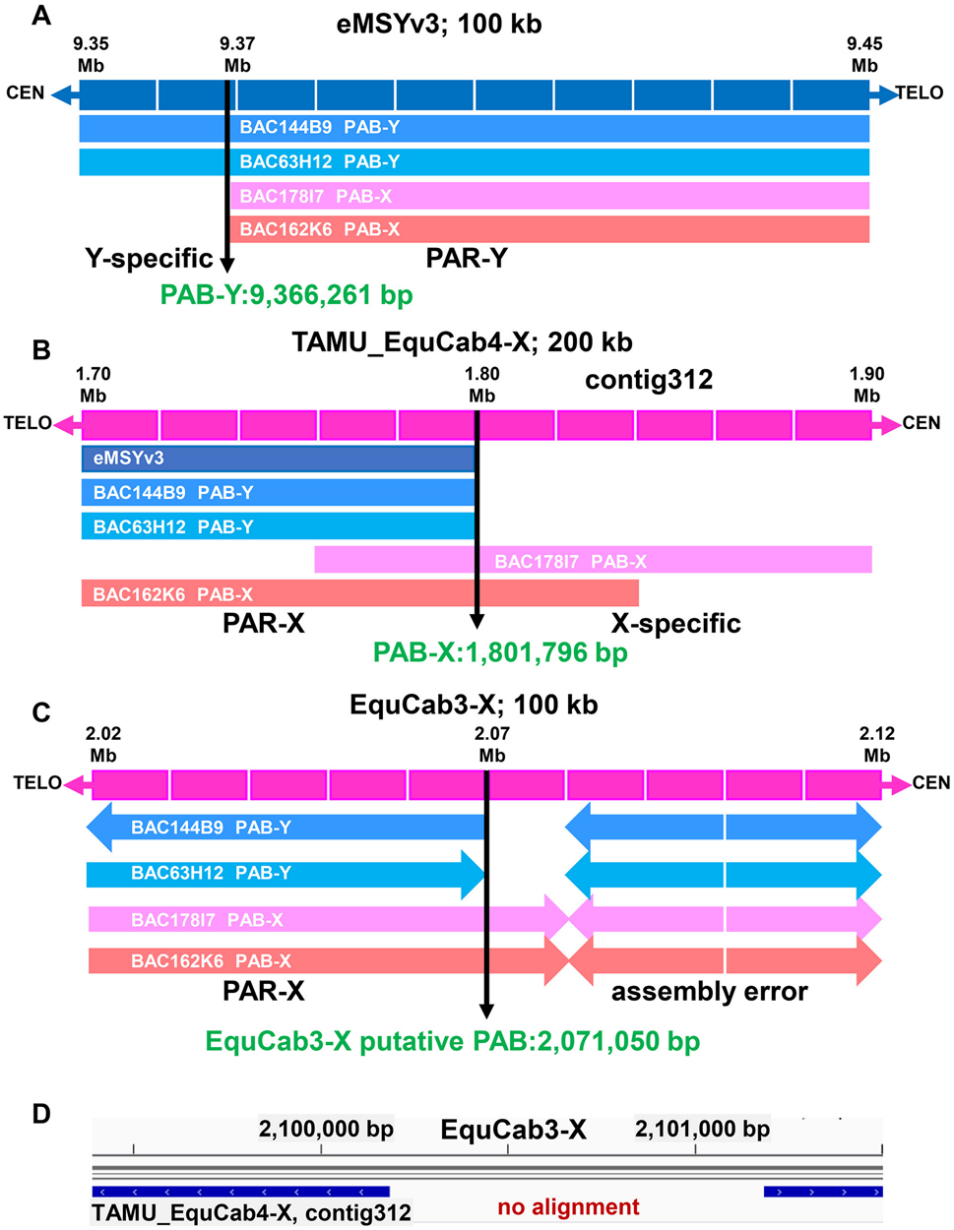
Identifying the horse PAB and the discovery of an assembly error in EquCab3 reference-X. (**A**) Stop of alignment of eMSYv3 with PAB-X BACs defines PAB-Y; (**B**) stop of alignment of eMSYv3 and PAB-Y BACs with TAMU_EquCab4-X defines PAB-X; (**C**) alignment of PAB-Y and PAB-X BACs (showing sequence orientation) with EquCab3-X near PAB reveals spurious duplication and inversion in reference X, and (**D**) alignment of TAMU_EquCab4-X contig 312 that contains the PAB shows no alignment with the erroneously assembled region in EquCab3-X; alignments were visualized in IGV^68^.

A prior study identified bacterial artificial chromosome (BAC) clones from the CHORI-241 library (https://bacpacresources.org/) that span the horse PAB in the Y chromosome (144B9 and 63H12) and in the X chromosome (178I7 and 162K6)^36^. Sequence alignment of these clones revealed two distinct alignment patterns—the PAR, where all four clones shared over 99% identity and sex chromosome specific regions, where similarity between PAB-X and PAB-Y BACs dropped dramatically. The latter indicated the location of the equine PAB with a landmark short interspersed nuclear element (SINE) MIR on the PAR side in both sex chromosomes (Fig. S1). Alignment of PAB BAC sequences with equine male-specific region of Y reference eMSYv3^37^, mapped PAB-Y at 9,366,261 bp—the position where the alignment with PAB-X BACs stopped but continued with PAB-Y BACs (Fig. 2A, Fig. S2A). Likewise, alignment of eMSYv3 and PAB BAC sequences with the newly built TAMU_EquCab4-X mapped PAB-X at 1,801,796 bp in the X chromosome—the position where the alignment of MSY and PAB-Y BACs stopped but continued with PAB-X BACs (Fig. 2B, Fig. S2B). Because TAMU_EquCab4-X contains the entire PAR, mapping PAB-X determined, for first time, the size of the horse PAR to be 1.8 Mb. However, when the PAB-spanning BACs were aligned with the current EquCab3-X reference, alignment with both PAB-Y BACs stopped at 2,071,050 bp but had a duplicated and inverted alignment in the X-specific region, proximal to PAB (Fig. 2C, Fig. S2C). Duplication and inversion at the same position was also observed with PAB-X BACs suggesting an assembly error in EquCab3-X. This was further confirmed by showing that primers designed from the duplicated/inverted region in EquCab3-X did not amplify by PCR and that the trio-binned contig from this region (contig 312) did not align with the inversion breakpoint in EquCab3-X (Fig. 2D, Fig. S2D).

**Figure 3:**
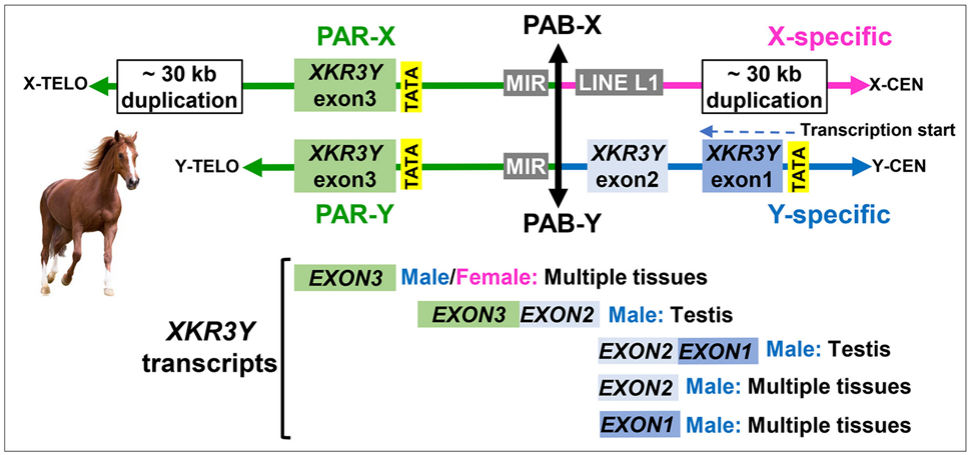
Annotation and transcriptional profile of the horse PAB and *XKR3Y*. Schematic drawing of the region around horse PAB in both sex chromosomes showing the relative position of *XKR3Y* exons, functional elements, and other landmarks (not drawn to the scale); PAR-Y is presented shorter because of incomplete assembly in eMSYv3; an arrow above *XKR3Y* exon1 shows the start and direction of transcription; transcripts revealed by RT-PCR are shown below *XKR3Y* exons. Horse image purchased from Bigstock (https://www.bigstockphoto.com/).

Next, we inspected sequences surrounding the PAB-X and PAB-Y for genes and other landmark features. The most distal annotated gene in the horse Y chromosome assembly^37^ is *XKR3Y*—an autosomal transposed gene that shares sequence similarity with XK family of membrane transport proteins^37^. The gene has three exons and the position of PAB-Y at 9,366,261 bp indicated that *XKR3Y* spans the PAB in the horse Y chromosome, so that exon1 and exon2 are in Y-specific region and exon3 is in the PAR (Fig. 3). This was confirmed by PCR showing that exon1 and exon2 were male-specific, and exon3 was present in both males and females (Fig. S3). Annotation of *XKR3Y* in eMSYv3^37^ shows that transcription starts from exon1 in the Y chromosome, which was confirmed by open reading frame (ORF) analysis of *XKR3Y* long IsoSeq transcripts. Transcriptional profiling of *XKR3Y* showed that exon1 and exon2 are transcribed in testis and several male somatic tissues but not in any female tissues (Fig. 3, Fig. S4). Two transcripts, one comprised of exons 1 and 2, another of exons2 and 3, were present only in testis. However, surprisingly, exon3 was transcribed in both male and female tissues (Fig. 3, Fig. S4). Hence, we searched the 1498 bp region between the PAB and exon3 for additional promoters and found TATA boxes 35–36 bp upstream of exon3 in both sex chromosomes (Fig. 3, Table S2). The presence of an intra-genic promoter explained how exon3 can be expressed from the X chromosome and suggested that exon3 can be expressed independently from exons1 and 2 in the Y chromosome.

In addition, we identified a ~ 5 kb LINE L1 element proximal to the PAB in the X-specific region, followed by a ~ 30 kb sequence that was duplicated in the PAR of TAMU_EquCab4-X (Fig. 3). No LINE L1 was present in the corresponding Y-specific region, suggesting that retrotransposition in the X chromosome may have contributed to the stop of X–Y recombination. The sequence that was duplicated in X-specific region and PAR-X was not found in PAR-Y because the assembly of PAR-Y in eMSYv3 is incomplete^37^. The duplicated 30 kb segment in the X chromosome was the likely cause of an assembly error in this region in EquCab3-X reference.

### The donkey pseudoautosomal boundary (PAB)

**Figure 4:**
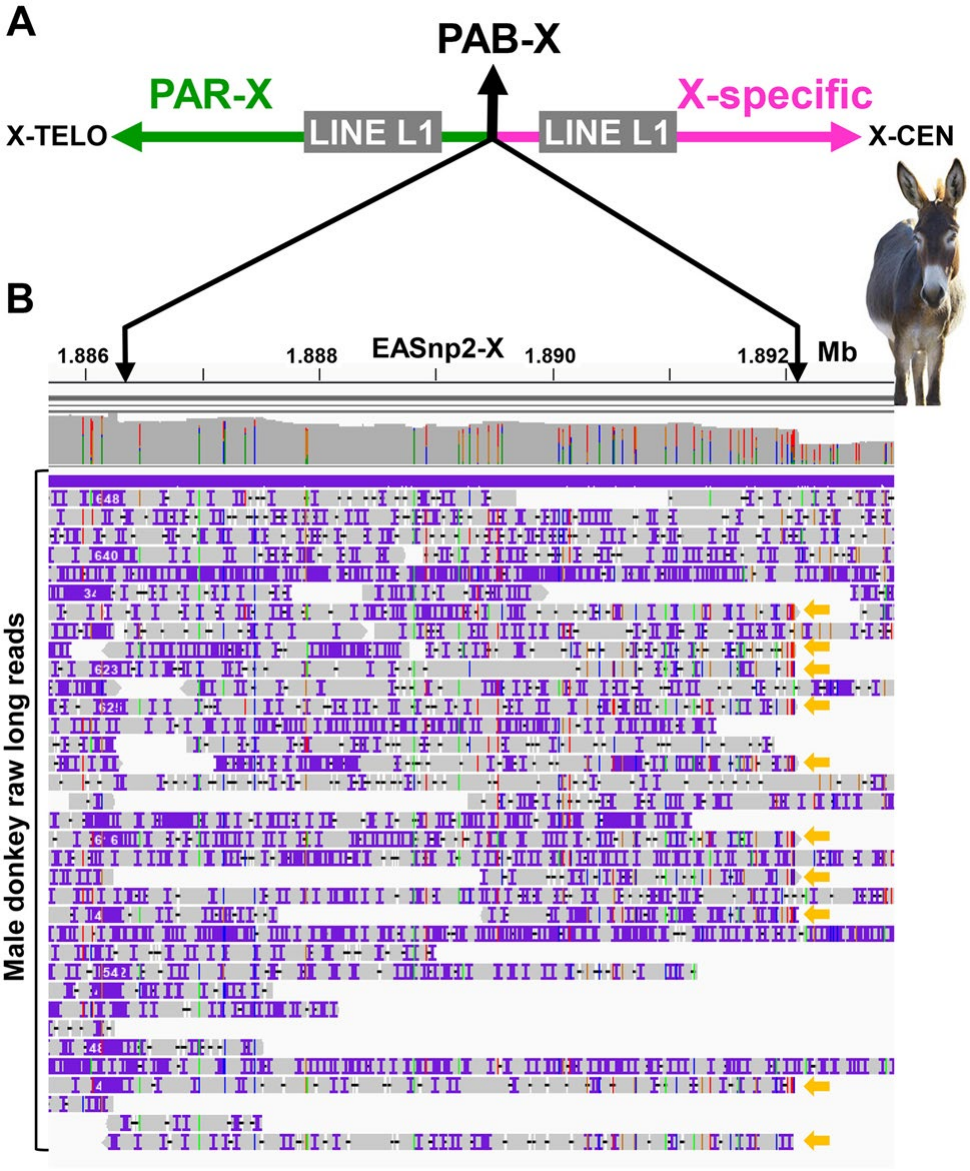
Donkey PAB. (**A**) Schematic drawing of the location of donkey PAB-X at 1,887,889 bp flanked by LINE L1 elements; (**B**) Alignment (in IGV^68^) of male donkey long reads (horizontal lines) to a ~ 6 kb region (demarcated by black vertical arrows) around PAB-X showing the start (left arrow) of drop in read coverage, reaching to 50% (right arrow) in the end; 10 male reads that are denoted with orange horizontal arrows are likely from the donkey Y chromosome because they drop off from the X chromosome at the same sequence position. Donkey image purchased from Bigstock (https://www.bigstockphoto.com/).

PCR analysis with horse PAB-X and PAB-Y BAC end sequence (BES) primers in male and female donkeys showed the same amplification patterns as were observed for male and female horses (Fig. S3), suggesting that the donkey PAB lies in the same ~ 200 kb sequence interval as the horse PAB. Alignment of TAMU_EquAsi2-X with horse eMSYv3 and PAB-spanning BACs mapped donkey PAB-X tentatively at 1,887,889 bp—the position where X and Y alignments stopped. Though, the alignment of horse PAB BACs with donkey X was more fragmented compared to horse-horse alignments, suggesting that sequences around the PAB differ between the two equids. Alignment of male donkey raw long reads^34^ with TAMU_EquAsi2-X revealed a ~ 6 kb region (from ~ 1.886 to ~ 1.892 Mb) where read coverage started gradual drop, reaching approximately 50% in the end (Fig. 4). This suggested that, in contrast to the horse PAB where X–Y sequence homology dropped abruptly (Fig. S1), homology between donkey PAR-X and PAR-Y sequences declines gradually. Though, we could not verify this or map donkey PAB-Y, because the donkey Y chromosome assembly in EquAsi1^34^ was not reliable (Table S7). However, perhaps the most interesting discovery was that the donkey *XKR3Y* gene was entirely Y-specific and not present in the X chromosome, as revealed by sequence alignments and exon-specific PCRs (Fig. S3). Other features of the donkey PAB-X were that there was no SINE MIR element near donkey PAB as it was in horse, and there were LINE L1 elements on both sides of donkey PAB-X. We concluded that even though horse and donkey PABs are in the same ~ 200 kb sequence interval, donkey PAR is ~ 86 kb larger than the horse PAR and the sequence landscape surrounding the PAB-X in the two species is different.

### Characterization of horse and donkey* DXZ4* macrosatellite repeats

The *DXZ4* macrosatellite tandem repeat has not been annotated in the EquCab3-X reference. While *DXZ4* sequences differ between species, the location of these repeats between the *PLS3* and *AGTR2* genes in the mammalian X chromosome is conserved^32, 38^. To evaluate the assembly of this structurally complex region in TAMU_EquCab4-X and TAMU_EquAsi2-X, we identified repeated sequences in this region of TAMU_EquCab4-X, though not in EquCab3-X where these sequences had collapsed (Fig. S5). The largest repeat unit was ~ 8 kb and the TAMU_EquCab4 had 9 full copies and two partial copies of *DXZ4* spanning ~ 71 kb. In contrast, only one full copy and three partial copies were found in EquCab3 (Table S3).

Using horse reference copy, 8 full and 3 partial copies of *DXZ4* were found in TAMU_EquAsi2-X spanning ~ 64 kb (Table S3). An additional 12 full and 2 partial copies of *DXZ4* were found in donkey scaffold 127 which was not included in the final assembly because of a small size (98 kb; size cut off for inclusion was 1 Mb). Though, this scaffold aligned to the horse assembly TAMU_EquCab4-X and likely belonged to the donkey X chromosome, thus, bringing the number of *DXZ4* full copies in the donkey to 20. Yet only four partial *DXZ4* copies with gaps were present in EquAsi1-X, consistent with our other observations about the poor quality of EquAsi1-X.

The CTCF (CCCTC-binding factor) binding site, a characteristic functional feature of the *DXZ4* repeat^38–40^, was observed three times in each *DXZ4* monomer and was the same in horse and donkey. Sequence similarity between *DXZ4* full copies of horse and donkey was 96%.

### Horse and donkey* ETSTY7* ampliconic array

**Figure 5:**
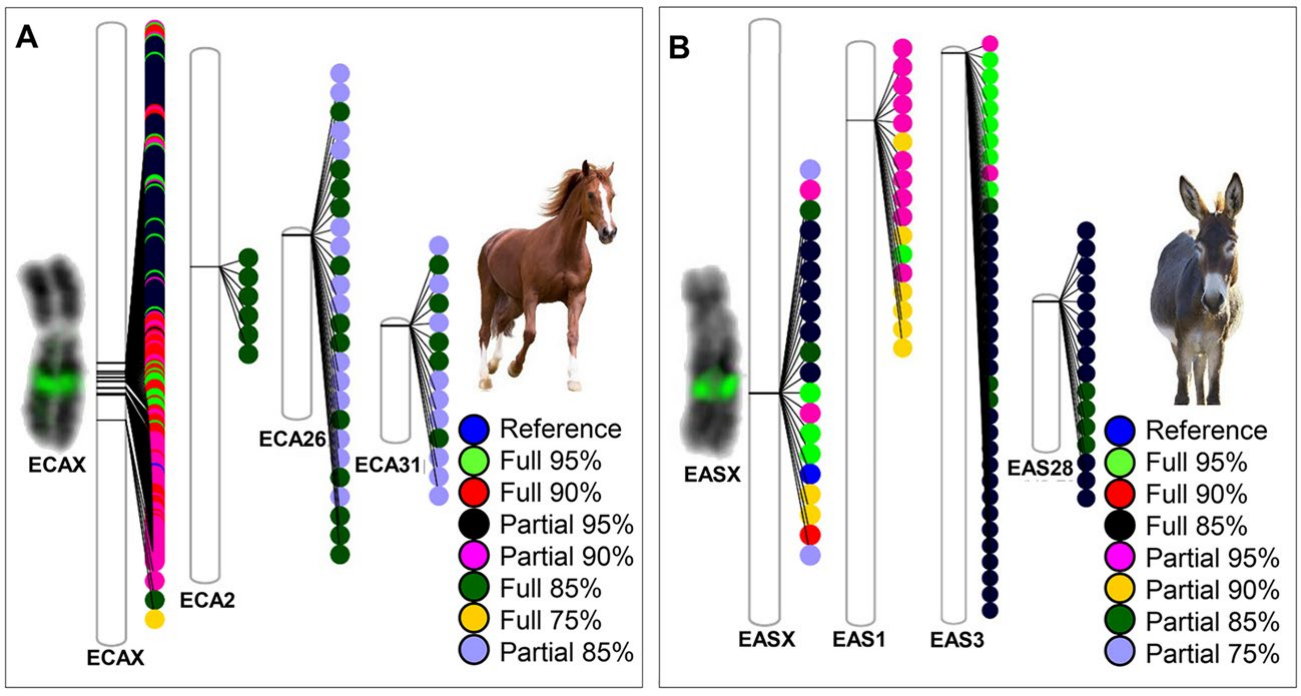
Distribution of *ETSTY7* full and partial amplicons in (**A**) horse (ECA) and (**B**) donkey (EAS) X chromosomes and autosomes using PhenoGram^70^; to the left of X chromosome phenograms are microscope images with inverted DAPI staining of ECAX and EASX showing the location of *ETSTY7* array (green) by FISH in the middle of the long arm (Xq). FISH data was generated by the authors and initially reported in Ref.^37^.

*ETSTY7* is a testis transcribed massively amplified array that has been assigned to equid (horse, donkey, and zebras) sex chromosomes and some autosomes (zebras only) by fluorescence in situ hybridization (FISH)^37^. Due to complexity, the sequence is partially assembled and annotated only in horse Y chromosome^37^. Alignment of *ETSTY7* reference copy from the Y chromosome (*ETSTY7*-Y) with TAMU_EquCab4-X, identified *ETSTY7* copies in three of the five X chromosome scaffolds (Table 1). Two of these, scaffolds 100032 and 100053, also aligned with EquCab3-X, while scaffold 100051 (over 9 Mb) was unique to TAMU_EquCab4. Using the top hit with *ETSTY7*-Y in the X chromosome as *ETSTY7*-X reference, 238 full and partial copies of *ETSTY7* were found in the X chromosome, and the majority had over 90% similarity with the X reference. A novel finding was that *ETSTY7* sequences were also present in some horse (*Equus caballus*, ECA) autosomes: 6 full copies in ECA2, 26 full and partial copies in ECA26, and 14 full and partial copies in ECA31 (Fig. 5A). Due to relatively low copy number, these regions were not previously detected by FISH analysis^37^. All autosomal *ETSTY7* copies had less than 85% similarity with the *ETSY7*-X reference and all *ETSTY7* autosomal arrays clustered near centromeres (Fig. 5A).

Donkey *ETSTY7* sequences in TAMU_EquAsi2-X were identified using horse *ETSTY7*-X reference and the top hit was then used as a reference for the donkey *ETSTY7*-X. The two references aligned 100% coverage with 94% identity. In contrast to hundreds of *ETSTY7* copies in the horse X chromosome, only 20 copies (12 full and 8 partial) were found in the donkey X (Fig. 5B). However, 3689 full or partial *ETSTY7* copies were present in 256 small (below the 1 Mb cut off for inclusion) unassigned donkey scaffolds. Since FISH results show that the size of *ETSTY7* ampliconic array in the horse and donkey X chromosomes is similar (Fig. 5), it is likely that many of these donkey unassigned scaffolds with *ETSTY7*, belong to the X chromosome, possibly into the gap that was visible in the alignment of TAMU_EquAsi2-X with TAMU_EquCab4-X (Fig. 1D).

Like in the horse, *ETSTY7* sequences were also present near centromeres of three donkey (*Equus asinus*, EAS) autosomes: 17 copies in EAS1, 36 copies in EAS3, and 16 copies in EAS28 (Fig. 5B). Notably, the regions containing *ETSTY7* arrays in EAS1 and EAS3, share conserved synteny with horse chromosomes, ECA31 and ECA2, respectively^41^. Though, there is no known Zoo-FISH homology between EAS28 and ECA26.

Finally, homology search for horse and donkey *ETSTY7*-X sequences in GenBank (https://www.ncbi.nlm.nih.gov/genbank/) did not add any new information to what is already known: the only sequences similar to equids *ETSTY7* was from the intestinal parasite *Parascaris,* thus consistent with the theory of horizontal transfer^37^.

### Horse and donkey haploid autosomal assemblies

While the focus of this study was on horse and donkey X chromosomes, significant byproducts of trio-binning of a hinny were improved autosomal assemblies of the two species (Table S4). Because of the high quality of EquCab3 reference^33^, improvements to the horse autosomal genome in TAMU_EquCab4 were relatively minor (Fig. S6, Table S5). Most horse autosomes were complete compared to EquCab3, except ECA18 and ECA29. In the trio-binning assembly, ECA18 was missing a 5–8 Mb proximal segment and ECA29, a 2–3 Mb segment from the 5′ end (Fig. S8). Both segments are present in EquCab3 but have highly discordant BAC tracks in NCBI Genome Data Viewer (https://www.ncbi.nlm.nih.gov/genome/gdv/) (Fig. S8), suggesting a mis-assembly in EquCab3, now corrected by trio-binning. It is worth mentioning that most acrocentric horse chromosomes, except ECA18 and ECA19, were assembled as a single scaffold, whereas bi-armed chromosomes remained in two or more scaffolds (Table S5). This indicates that even the long DNA molecules that were used for optical mapping could not span centromeric satellite or other complex repeats while scaffolding. Unsurprisingly, the only bi-armed chromosome that assembled into a single scaffold was ECA11, which is known to have a satellite-free centromere^42^. On the other hand, the most fragmented autosomes, with four scaffolds each, were ECA9 and ECA12 (Table S5), likely due to their known structural complexity: ECA9 contains large number of *LCORL* retrocopies^43^ and ECA12 is highly enriched with copy number variable olfactory receptor gene clusters^44^.

In contrast to the horse, the new donkey autosomal assembly, TAMU_EquAsi2, presents a dramatic improvement over EquAsi1 reference^34^ by including ~ 60 Mb of missing genomic sequence (Tables S4, S6) and correcting innumerous assembly errors (Fig. S7, Table S7). Of the 30 donkey autosomes, assemblies of only 13 chromosomes were more or less consistent between EquAsi1 reference^34^ and TAMU_EquAsi2 as well as with Zoo-FISH-based^41^ homologous regions in EquCab3. The remaining 17 donkey autosomes showed extensive differences between the trio-binning assembly and the EquAsi1 reference, bringing into question the reliability of the latter (Fig. S7, Table S7). As a result of trio-binning assembly, we reassigned interchromosomally approximately 231 Mb of sequences (~ 9% of the 2.5 Gb genome) that were placed on wrong chromosomes in EquAsi1. Additionally, we corrected multiple intrachromosomal discordances. Despite this progress, the final scaffold level assemblies of donkey autosomes remained fragmented with only 12 autosomes assembled into a single scaffold (Table S6). Among these, EAS7, EAS9, EAS13, and EAS19 are bi-armed chromosomes with satellite-free centromeres^45^, like ECA11 mentioned above. Though, most other donkey bi-armed autosomes with satellite-free centromeres (EAS4, 5, 8, 10, 11, 12, 13, 14, 16, and 18) remained in two or more scaffolds, indicating that centromeric repeats are not the only sequences that complicate chromosome assemblies.

## Discussion

Here we report quantitative and qualitative improvement of the horse and donkey X chromosome assemblies using long read sequences from a female hinny—a hybrid of a horse stallion and a jenny donkey. Both chromosome assemblies were longer—horse by 15 Mb and donkey by18 Mb, and more accurate as we corrected errors in current references and resolved *DZX4* macrosatellite repeats and a portion of *ETSTY7* amplicons. In addition, improved donkey X assembly allowed, for the first time, to demarcate the ~ 18 Mb pericentric inversion at the sequence level (Fig. S2) that distinguishes donkey X chromosome from the X chromosomes of other equids^35^. The distal inversion breakpoint at ~ 31 Mb in the horse X is consistent with prior FISH data that mapped the start of the inversion distal to the *OTC* gene^35^ (EquCab3-X: *OTC* at 32 Mb). Assignment of the proximal breakpoint at ~ 49 Mb in the horse X chromosome, however, is more approximate and requires better assembly of the pericentromeric region (Fig. S2).

Despite this progress, the two X chromosomes are still not complete as both are comprised of multiple scaffolds (Table 1, Tables S5, S6) and have gaps of unknown size and contents. On the other hand, the 143.2 Mb size of TAMU_EquCab4-X (Table 1) is close to the 146 Mb of cattle X chromosome that was recently improved by trio-binning of Brahman and Angus breeds^46^ and may approach the 154.3 Mb of gapless T2T human X chromosome when those methods are utilized in these species^21^. It is likely that the horse X is only missing centromeric repeats and a few complex regions in chromosome arms. Since horse and donkey X chromosomes are cytogenetically of similar size (Figs. 1D, 5), more sequences are missing from the 129.1 Mb TAMU_EquAsi2-X. Given that the donkey X chromosome centromere is satellite-free^45^, the gaps are likely in chromosome arms, e.g., hundreds of missing *ETSTY7* amplicons in Xq (Fig. 1). Lessons from T2T assembly of the human X chromosome^21^ and autosomes^28^ show that the only way to resolve such complex sequences is to produce ultra-long (Oxford Nanopore) reads across these regions and use high fidelity long (PacBio HFi/CCS) and short (Illumina) reads for polishing^21^.

An important outcome of the present study was mapping the horse and donkey PAB and demarcating the PAR at sequence level. In fact, horse was the first non-primate/non-murine eutherian species where the approximate location of the PAB and a tentative size of the PAR were determined from a BAC tiling path^36^ before the release of the first reference genome EquCab2^42^. In EquCab2-X, though, the PAR was incomplete, had multiple gaps, assembly errors, and missed the terminal ~ 700–800 kb segment. The missing end of the PAR was included, and most gaps were closed in the next reference EquCab3-X^33^, but there remained critical errors in the assembly that prevented delineating the PAB. These errors were corrected in TAMU_EquCab4-X (Fig. 2C,D) in this study and the size of the horse PAR was determined to be 1.8 Mb, exactly as proposed from PAR BAC tiling path 15 years ago^36^. Similar size, 1.88 Mb, was estimated for the donkey PAR (Fig. 4), which is consistent with earlier observations that the size of the PAR and the location of the PAB in all equids are similar^47^. These findings also confirmed that horse/donkey/equids PAR is among the smallest known in eutherian mammals, being larger only from the ~ 700 kb mouse PAR^48^ but smaller than the ~ 2.7 Mb size PAR1 in humans and simian primates^1, 47^. In all other eutherians where the PAR has been demarcated, the region is magnitudes larger, around 6–9 Mb^47^. For example, the recent completely assembled cattle PAR is 6.84 Mb and comparative gene maps suggest that the PAR is highly collinear and of similar size also in goat, sheep, river buffalo, dog, and pig^46^. This is important because PAR size may have implications on sex chromosome pairing and segregation in male meiosis and consequently, on the occurrence of sex chromosome aneuploidies^49^. Because PAR genes escape X inactivation^14, 15^, the PAR size and gene content are critical for understanding phenotypic consequences of gene overdose in females or haploinsufficiency resulting from these aneuploidies^17, 47^. This is consistent with the highest reported incidence of viable X-monosomy in horses and humans, the species with small PARs^50^. No such information is available for other equids.

Despite similar size and overall sequence alignment of the horse and donkey PARs, the regions around their PABs delineated surprisingly differently (Figs. 3, 4). Of particular interest was the *XKR3Y* gene, which spans the PAB in the horse Y chromosome (Fig. 3) but is entirely Y-specific in the donkey (Fig. S3). If this difference resulted from an evolutionary movement of donkey PAB towards the distal end of the Y chromosome, donkey PAR should be shorter. Our results showed the opposite—donkey PAR was 1.88 Mb and horse PAR 1.8 Mb, suggesting that there are more rearrangements in the PAB region between the two equids. Detailed characterization of these differences, however, requires a more reliable donkey Y assembly than is currently available. It is possible that the divergence of PAB sequences and different sex-linked status of *XKR3Y* in horses and donkeys, contribute to the reproductive barrier and sterility of hybrid males. This is in line with RNAseq data showing that *XKR3Y* is the most significantly downregulated (395-fold) gene in mule testis, compared to horse and donkey testis^37^. While these findings and testis-predominant transcriptional profile of *XKR3Y* (Fig. S4) suggest a role in spermatogenesis, not much is known about the functions of this gene in any species, except that the expression of the closest non-equid ortholog, the human autosomal *XKR3*, is even more strictly limited to testis (NCBI Gene: https://www.ncbi.nlm.nih.gov/gene/150165/). Finally, it is worth mentioning that while there are distant orthologs of horse *XKR3Y* in the Y chromosome of several mammals^37^ the X-linked *XKR3Y* is found, so far, only in the horse (though poorly annotated and denoted as LOC100064929 in EquCab3). This also means that, while the gene map of the horse PAR is quite collinear with human and many other eutherian X chromosomes^47^, the region around horse PAB-X is not. Another unusual feature of the horse PAB is that it truncates a gene in the X chromosome. In all other species where the PAB is annotated and spans a protein coding gene, like *XG* in human^51^, *MID1* in mouse^52^, *GPR143* in cattle^53^, and *SHROOM2* in pig^54^, the gene is truncated in the Y chromosome and remains intact in the X^47^. Consequently, in these species the PAB-spanning genes can be equally expressed in males and females, while the equine *XKR3Y* is expressed in full only in males (Fig. S4), which is an additional support for its putative role in male fertility or hybrid male sterility.

In addition to mapping and characterization of the horse and donkey PAB, the improved assemblies of the X chromosomes allowed for the resolution of a functionally important repetitive region corresponding to *DXZ4* macrosatellite. The organization of this tandem array has been characterized in human and primates^55, 56^, mouse^38^, and recently in felids^4^. It appears that the only conserved features across species are the location of *DXZ4* between the *PLS3* and *AGTR2* genes, and the presence of CTCF binding domains. Otherwise, the *DXZ4* sequence, the size of a monomer, extent of sequence variation between monomers, and the number of monomers vary greatly between species. Human *DXZ4* contains 12 to 100 monomers of very similar length (3 kb)^55^; murine *Dxz4* is composed of 7 monomers of varying length (3.8 and 5.7 kb)^38^, and feline *DXZ4* has a unique compound structure and is copy number variable between related species^4^. Here we provided the first characterization of the horse and donkey *DXZ4,* showing that the sequence is similar between the two species, the length of the most predominant monomer is ~ 8 kb, and that there were 10 *DXZ4* copies in the horse and 20 in the donkey (Table S3). While the details of equid *DXZ4* array organization are yet to be determined, these preliminary data provide a necessary foundation for proper annotation of this region in genome assemblies of individual horses, donkeys, and other equids, determine the range of *DXZ4* structural and copy number variation between individuals and species, and study its functional features. The latter is of particular interest because, *DXZ4* is essential for X chromosome inactivation^39^ and possibly also for meiotic sex chromosome inactivation and a strong candidate locus for hybrid male sterility^4^.

More enigmatic is the genetic role of the *ETSTY7* ampliconic array which has been partially (15 copies, 3 exons each) assembled in eMSYv3^37^ and was now also included in the assembly of the horse and donkey X chromosomes. Consistent with FISH analysis^37^, this testis-transcribed sequence had hundreds of copies in both X chromosomes (Fig. 5) and, therefore, contributed most to the increased size of TAMU_EquCab4-X and TAMU_EquAsi2-X. A novel finding, though, was assigning a smaller number and more diverged *ETSTY7* copies to three horse and three donkey autosomes. The fact that two autosomes (ECA31-EAS1 and ECA2-EAS3; Fig. 5) are homologous by Zoo-FISH^41^ supports the theory that the *ETSTY7* transcript family was acquired before equids split 4.0–4.5 million years ago^57^. There is compelling evidence that the sequence originates from an intestinal parasite, *Parascaris equorum*, by horizontal transfer^37^ because no similar sequences can be found in any other eukaryotic species. This also hinders using model species to investigate *ETSTY7* functions. It is possible that *ETSTY7* manifests sex-linked meiotic drive, as it is most abundantly amplified in horse X and Y chromosomes, though not detected in the donkey Y chromosome by FISH^37^. This may be due to less *ETSTY7* copies and limited sensitivity of FISH, as was the case of finding horse and donkey autosomal *ETSTY7* by sequence analysis in this study and not previously by FISH^37^. In mammals, similar sex-linked and lineage-specific amplicons with known or predicted role in meiotic drive, have been observed in mouse and some other rodents^6, 7^, cattle^5^, and cat and dog^58^. In this context, the persistence of autosomal *ETSTY7* amplicons remains unclear.

Finally, in addition to the haploid assemblies of the horse and donkey X chromosomes, the trio-binning approach also produced haploid assemblies for all horse and donkey autosomes. Considering that the average identity between the two genomes, as well as the X chromosomes separately, was ~ 98% and only 94% for the *ETSTY7* sequences, we are confident that the haploid genomes of the horse and the donkey, diverged for about 4 million years ago^59^, were separated accurately. This is consistent with the first trio-binning study using CANU to successfully separate 99.35% identical haplotypes of the Angus and the Brahman cattle^23^, which diverged only 250,000 years ago^60^. The new haploid assemblies in this study improved the horse and donkey autosomes, whereas the improvements for donkey chromosomes were extensive compared to the current chromosome-level reference EquAsi1^34^ (Table S7). Despite this, many complex sequences including centromeric satellite repeats, remained unassigned in both species as evidenced from fragmented multi-scaffold assemblies of many autosomes. Obviously, the use of PacBio long reads and scaffolding with optical map long molecules only, is not sufficient to resolve all complex genomic sequences. The latter require a combination of approaches, including high-coverage ultra-long Oxford Nanopore reads, that have led to the T2T assemblies of all human autosomes^28^ and the sex chromosomes^21, 29^.

In conclusion, the improvements that were made for the horse and donkey X chromosomes are expected to advance the study of X-linked conditions, X chromosome regulation, meiotic behavior of the sex chromosomes, and sex chromosome evolution in equids. Likewise, the important additional products of this study—the more accurate, more complete, and contiguous assemblies of horse and donkey autosomes (Figs. S6, S7, Tables S5–S7), contribute to molecular studies of equid biology and evolution.

## Materials and methods

### Ethics statement

Procurement of samples followed the United States Government Principles for the Utilization and Care of Vertebrate Animals Used in Testing, Research and Training. These protocols were approved as IACUC #2018-0342 CA and IACUC #1986-0216 at Texas A&M University and Cornell University, respectively. All methods are reported in accordance with ARRIVE guidelines (https://arriveguidelines.org) for the reporting of animal experiments.

### Animals, samples, and DNA

Peripheral blood in EDTA vacutainers (VACUTAINERTM, Becton Dickinson) was obtained from a female hinny *#3742,* housed at Cornell University. Archived frozen DNA samples were available from the hinny’s parents—a Thoroughbred stallion *#3105* and a jenny donkey *#3524*. Primary fibroblast cell line in liquid nitrogen was available for a Thoroughbred stallion *Bravo,* the DNA donor for the CHORI-241 horse BAC library (https://bacpacresources.org/) and for the horse Y chromosome reference assembly^37^. The hinny’s sire *#3105* was also the sire of *Bravo*, as well as the sire of *Twilight*, the DNA donor for the horse reference genomes EquCab2^42^ and EquCab3^33^. High molecular weight (HMW) genomic DNA (gDNA) was extracted from hinny’s blood and the fibroblasts of the Thoroughbred stallion *Bravo* using MagAttract HMW DNA kit (Qiagen) following manufacturer’s protocol. DNA quality and fragment size were checked by pulsed-field gel electrophoresis (PFGE) showing most DNA fragments being between 50 and 100 kb.

### Sequencing the trio

High molecular weight gDNA of the hinny was used for PacBio (Pacific Biosciences) long-insert, size-selected library preparation and sequenced across three PacBio Sequel II 8 M SMRT cells. Genomic DNA samples from hinny’s horse sire and donkey dam were used to prepare 450 base-pair (bp) fragment size Illumina libraries and sequenced as 2 × 150 bp on Illumina NovaSeq 6000 S4 platform to approximately 30X genome coverage.

### Generation of Hi-C and optical genome map data for scaffolding

Horse Hi-C libraries were made from the primary fibroblast cultures of the Thoroughbred stallion *Bravo* using Arima-HiC kit (Arima Genomics) following the manufacturer’s instructions for mammalian cell lines. Libraries were sequenced on the Illumina NovaSeq6000, yielding approximately 1.5 billion paired end reads (180X Coverage). Donkey Hi-C data were retrieved from NCBI Sequence Read Archive (SRA: https://www.ncbi.nlm.nih.gov/sra; SRX9609014) and from DNAzoo (https://www.dnazoo.org/; ASM303372). Optical genome maps (OGM) for the horse and donkey were generated with Bionano Saphyr system proprietary protocols, kits, equipment, and software packages (Bionano Genomics). As we did not have HMW DNA for the sire and dam of the hinny, we used HMW gDNA from the closely related Thoroughbred stallion *Bravo* for horse and donkey #3611 provided by Cornell University to generate OGM. Briefly: HMW gDNA was labeled with methyltransferase DLE-1 at the recognition motif CTTAAG using Bionano DLS DNA Labeling Kit, generating approximately 15 labels per 100 kb. Labeled DNA molecules were applied to Bionano G1.2 flow cells, linearized in nanochannels, and scanned with a fluorescence microscope. The captured images were converted to electronic representations of the DNA molecules.

### Sequence assembly

#### Contig-level assembly

Genome assembly was done in a stepwise manner. Initial contig-level assemblies were built with the trio-binning function of CANU with standard parameters^23, 61^ using short read data from Sequence Read Archive (SRA) for horse (SRX1485179) and donkey (ERX2338438)^59^.

#### Scaffold-level assembly

Initial scaffolding of horse and donkey contig-level assemblies was done with the Hi-C data using SALSA2 with the “-e GATC” command^62^. The Hi-C scaffolded horse assembly was further scaffolded with horse OGM data using the Bionano Solve pipeline by in silico digesting the Hi-C assembly using the DLE-1 hexamer and aligning it with the OGM data of Thoroughbred *Bravo*. The donkey contig-level assembly was scaffolded only with the OGM data in the same manner as horse.

#### Chromosome-level assembly

Horse and donkey scaffolded assemblies were aligned to horse and donkey reference genomes EquCab3 and EquAsi1, respectively, using NUCmer function of MuMmer^63^. The resulting delta files were uploaded into Assemblytics^64^ and the alignments were viewed through the interactive alignment viewer. Scaffolds corresponding to horse and donkey X chromosomes, as well as to 31 horse and 30 donkey autosomes were identified and realigned to their respective chromosome with Minimap2^65^. In donkey reference, EquAsi1^34^, assemblies of several chromosomes were not reliable as revealed by their misalignment to the known Zoo-FISH-based conserved synteny blocks^41^ in horse reference, EquCab3. In these cases, we identified donkey scaffolds according to Zoo-FISH data from EquCab3. Scaffolds corresponding to individual chromosomes were ordered and oriented, then concatenated and realigned with Minimap2 to the respective chromosomes. The alignments were viewed as dot plots for correctness.

#### Polished final assembly

The horse and donkey chromosome-level WG assemblies were polished with Illumina reads for the stallion sire and the jenny dam, respectively, using NextPolish^66^. The resulting assemblies were denoted as TAMU_EquCab4, for the horse and TAMU_EquAsi2, for the donkey. To determine whether polishing introduced any large-scale rearrangements, TAMU_EquCab4 and TAMU_EquAsi2 were aligned with their respective unpolished assemblies using Minimap2^65^ and visualized as dot plots.

### Demarcation of the pseudoautosomal boundary (PAB)

Sequences of horse BAC clones (CHORI-241: https://bacpacresources.org/resources.htm) known to span the horse PAB in the Y chromosome (PAB-Y: 63H12; NCBI #AC214633.5 and 144B9; NCBI #AC214971.2) and in the X chromosome (PAB-X: 162K6; NCBI #AC218091.1 and 178I7; NCBI #AC217547.2)^36^ were aligned with ClustalW (http://www.clustal.org/). To determine the location of the horse PAB, the BAC sequences were aligned by megaBLAST^67^ with horse X chromosomes in female genomes EquCab3 and TAMU_EquCab4 (this study), and with horse Y reference eMSYv3^37^. For the donkey PAB, horse PAB-Y and PAB-X BAC sequences were aligned with the X chromosome in TAMU_EquAsi2 (this study) by megaBLAST^67^ and with raw long reads from a male donkey^34^ (SRA: SRR7031465, SRR7031466, SRR7031494, SRR7031493, SRR7031492, SRR7031491, SRR7031490, SRR7031489, SRR7031488, SRR7031487, SRR7031496, SRR7031495) using the pb-raw option of Minimap2^65^. The alignment output .bam file was sorted and indexed with SAMtools, and uploaded into Integrative Genomics Viewer, IGV^68^ for visualization.

### Analysis of the *XKR3Y* gene

#### Primers and qualitative PCR

Primers were designed with PrimerQuest™ Tool (Integrated DNA Technologies, IDT) for the three exons of the horse *XKR3Y* gene—the most distal gene in the horse Y reference assembly eMSYv3^37^ (Table S1). Primers for the end of PAB-X and PAB-Y spanning BAC were retrieved from Ref.^36^. The primers were tested on male and female horse and donkey gDNA by regular qualitative PCR with 5xFIREPol® Master Mix (Solis BioDyne, Tartu, Estonia) and the products were resolved in 2% agarose gel with a 100 bp ladder (New England Biolabs, Ipswich, MA).

#### Reverse-transcriptase PCR (RT-PCR)

Normal adult male and female horse somatic tissues (brain, kidney, heart, skeletal muscle, liver, lung, and spleen), testis, and ovary were stored in RNA-later (Invitrogen) at − 80 °C. Total RNA was isolated with RNeasy Mini Kit (Qiagen) following the manufacturer’s protocol. The samples were treated with DNaseI (Ambion), quality checked with BioAnalyzer 2100 (Agilent) and quantified with a Nanodrop spectrophotometer. RT-PCR reactions were carried with horse *XKR3Y* primers for exons 1, 2, and 3, and their combinations (Table S1) in 15 μL volume using Superscript III One-Step RT-PCR System and Platinum TaqDNA polymerase (Invitrogen). The RNA samples were analyzed simultaneously with gDNA controls and a housekeeping gene *ACTB.*

#### Testis IsoSeq

RNA IsoSeq data was generated for adult horse testis on PacBio Sequel II platform and used to create an IsoSeq transcript BLAST database. Exonic sequences of *XKR3Y* were searched against this database by megaBLAST and *XKR3Y* transcripts were isolated from the original IsoSeq output FASTA file with faidx command of SAMtools.

#### Open reading frame (ORF) and promoter analysis

ORFs were determined with ORFfinder (NCBI: https://www.ncbi.nlm.nih.gov/orffinder/). Sequences upstream *XKR3Y* exon3 (in the PAR) were extracted from eMSYv3^37^ and TAMU_EquCab4-X (this study) with SAMtools faidx and manually searched for promoters by looking for the canonical sequences of a TATA box, TATAWAW, where W is either T or A^69^.

### Assembly and analysis of *DXZ4* macrosatellite repeat

*DXZ4* macrosatellite maps between *PLS3* and *AGTR2* in the X chromosome of human, felids and mouse^4, 38^. The corresponding region was extracted from EquCab3 with SAMtools faidx and aligned to TAMU_EquCab4-X with MashMap (D-GENIES). A *DXZ4* repeated unit was extracted from TAMU_EquCab4-X with SAMtools faidx and used as a reference to localize *DXZ4* in EquCab3-X and TAMU_EquCab4-X with both megaBLAST and Minimap2. Similarly, a reference copy for donkey *DXZ4* was identified and localized in TAMU_EquAsi2-X. The CTCF binding domain (AGTTTCGCTTGATGGCAGTGTTGCACCACGAAT)^4, 38^ was identified and analyzed by BLASTn.

### Assembly and analysis of* ETSTY7* ampliconic array

The genomic sequence of Equine Testis Specific Transcript in Y7, *ETSTY7,* was obtained from the horse Y reference eMSYv3^37^ and mapped to TAMU_EquCab4-X by megaBLAST. From multiple copies, the top hit was extracted with SAMtools faidx and used a reference copy to align with TAMU_EquCab4 and TAMU_EquAsi2 WG assemblies by megaBLAST. Only hits over 1 kb were considered. All copies of *ETSTY7* from the horse genome TAMU_EquCab4 were extracted with a custom BASH command utilizing SAMtools faidx and aligned with ClustalW (http://www.clustal.org/). A neighbor joining tree was built using standard parameters. The tree was not mid rooted as no outgroup was included. Chromosomal locations of *ETSTY7* full and partial copies were visualized with PhenoGram software^70^.

### Supplementary Information

Supplementary Figures.

Supplementary Tables.

## Data Availability

All data is available from NCBI under BioProject PRJNA988091 (Temporary Submission ID: SUB13504527). PacBio sequence data is accessioned as SAMN36340620. Illumina data for the sire and dam are SAMN36340622 and SAMN36340621, respectively. Thoroughbred stallion *Bravo* Hi-C data is available as SAMN36340623. Testis ISOseq data is deposited as SAMN36340624. SRA records will be accessible with the following link after publication: https://urldefense.com/v3/; https://www.ncbi.nlm.nih.gov/sra/PRJNA988091__;!!KwNVnqRv!G6SNXT6VsgLelS5e_izzhC0YrNoo5gyuBP_hV_Yjo5H14tsdNwbegS6NLPfHZpLy-xtHBgtl6D0veXJk3bMZGQ$.
