# Supplementary material for: Trio-binning of a hinny refines the comparative organization of the horse and donkey X chromosomes and reveals novel species-specific features
Source: Sci Rep. 2023 Nov 17;13:20180. doi: 10.1038/s41598-023-47583-x (PMC10656420; doi:10.1038/s41598-023-47583-x)
Supplement: Supplementary file 1 — Supplementary Figures. [file 41598_2023_47583_MOESM1_ESM.pdf]

Trio-binning of a hinny refines the comparative organization of the horse and donkey X chromosomes and reveals novel species-specific features

Matthew J. Jevit[1], Caitlin Castaneda[1], Nandina Paria[2], Pranab J. Das[3], Donald Miller[4], Douglas F. Antczak[4], Theodore S. Kalbfleisch[5], Brian W. Davis[1]*, and Terje Raudsepp[1]*

[1] School of Veterinary Medicine, Texas A&M University, College Station, TX 77843, USA
[2] Texas Scottish Rite Hospital for Children, Dallas, TX 75219, USA
[3] ICAR-National Research Centre on Pig, Rani, Guwahati, Assam 781131, India
[4] Baker Institute for Animal Health, Cornell University, Ithaca, NY 14853, USA
[5] Maxwell H. Gluck Equine Research Center, University of Kentucky, Lexington, KY 40546, USA

*Corresponding authors:
Terje Raudsepp traudsepp@cvm.tamu.edu
Brian W. Davis bdavis@cvm.tamu.edu

Figure S1. Sequence alignment of four horse BAC clones spanning the PAB: clones 178I7 and 162K6 span PAB in the X chromosome (PAB-X) and clones 144B9 and 63H12 span PAB in the Y chromosome (PAB-Y). A region where all four clones share over 99% sequence identity corresponds to the PAR (green box). A SINE element at the PAB is shaded gray. The lower three lines of alignment (blue box) correspond to sex chromosome specific sequences where sequence similarity between PAB-X and PAB-Y BACs drops dramatically. Note that in both regions, sequences of the two PAB-X BACs and the two PAB-Y BACs are 100% identical.

Figure S2. Pericentric inversion in the donkey X chromosome. Alignment dot plots (A) between EquCab3-X and TAMU_EquAsi2-X and (B) TAMU_EquCab4-X and TAMU_EquAsi2-X map the distal inversion breakpoint approximately to 31 Mb and the proximal breakpoint to approximately 49 Mb in the horse X chromosome. Note the reduced horse-donkey alignment accuracy in the proximal portion of the inversion (arrows), likely due to centromeres.

Figure S3. **PCR analysis of the horse and donkey PAB and the *XKR3Y* gene.** (**A**) PCR results on gDNA of male and female donkeys and horses using end sequence primers of the two BAC clones that span PAB in the horse Y chromosome (Raudsepp and Chowdhary, 2008), and (**B**) PCR results on gDNA of male and female donkey and horses using primers specific for the three exons of the horse *XKR3Y* gene.

Figure S4. Reverse transcriptase PCR on male (left) and female (right) somatic tissues, testis and ovary with horse *XKR3Y* exonic primers and their combinations showing that exons 1 and 2 are male-specific and exon 3 (PAR) expressed in male and female somatic tissues. Isoforms comprised of exons 1 and 2 and exons 2 and 3 are exclusively expressed in testis.

Figure S5. Mashmap alignment of regions surrounding *DXZ4*. EquCab3-*DXZ4* (x-axis) and TAMU_EquCab4-*DXZ4* (y-axis); a collapsed sequences in EquCab3-X are in pink box.

Figure S6. Alignment dot plots of all TAMU_EquCab4 (y-axis) and EquCab3 (x-axis) autosomes; the alignments were done with Minimap2 function of D-Genies.

Figure S7. Alignment dot plots of all TAMU_EquAsi2 (y-axis) and EquAsi1 (x-axis) autosomes; the alignments were done with Minimap2 function of D-Genies; detailed comments about assembly problems in EquAsi1 and corrections made in TAMU_EquAsi2 are listed in Table S7.

Figure S8. Corrections in horse autosomal assembly for ECA18 (A) and ECA29 (B). (A-left) Alignment of ECAnp4-18 (y-axis) to EquCab3-18 (x-axis). The red box marks the sequences present in EquCAb3-18 but not present in ECAnp4-18. **(A-right)** The missing region is present in EquCab3-18 viewed with the BAC clone tack. The red lines correspond to BACs which ends are discordantly mapped in this sequence indicating it may be assembled incorrectly. **(B-left)** Alignment of ECAnp4-29 (y- axis) to EquCab3-29 (x-axis). The red box marks the sequences present in EquCab3-29 but not present in ECAnp4-29. (**B-right)** The missing region is present in EquCab3-29 viewed with the BAC clone tack. The red lines correspond to BACs which ends are discordantly mapped in this sequence indicating it may be assembled incorrectly.

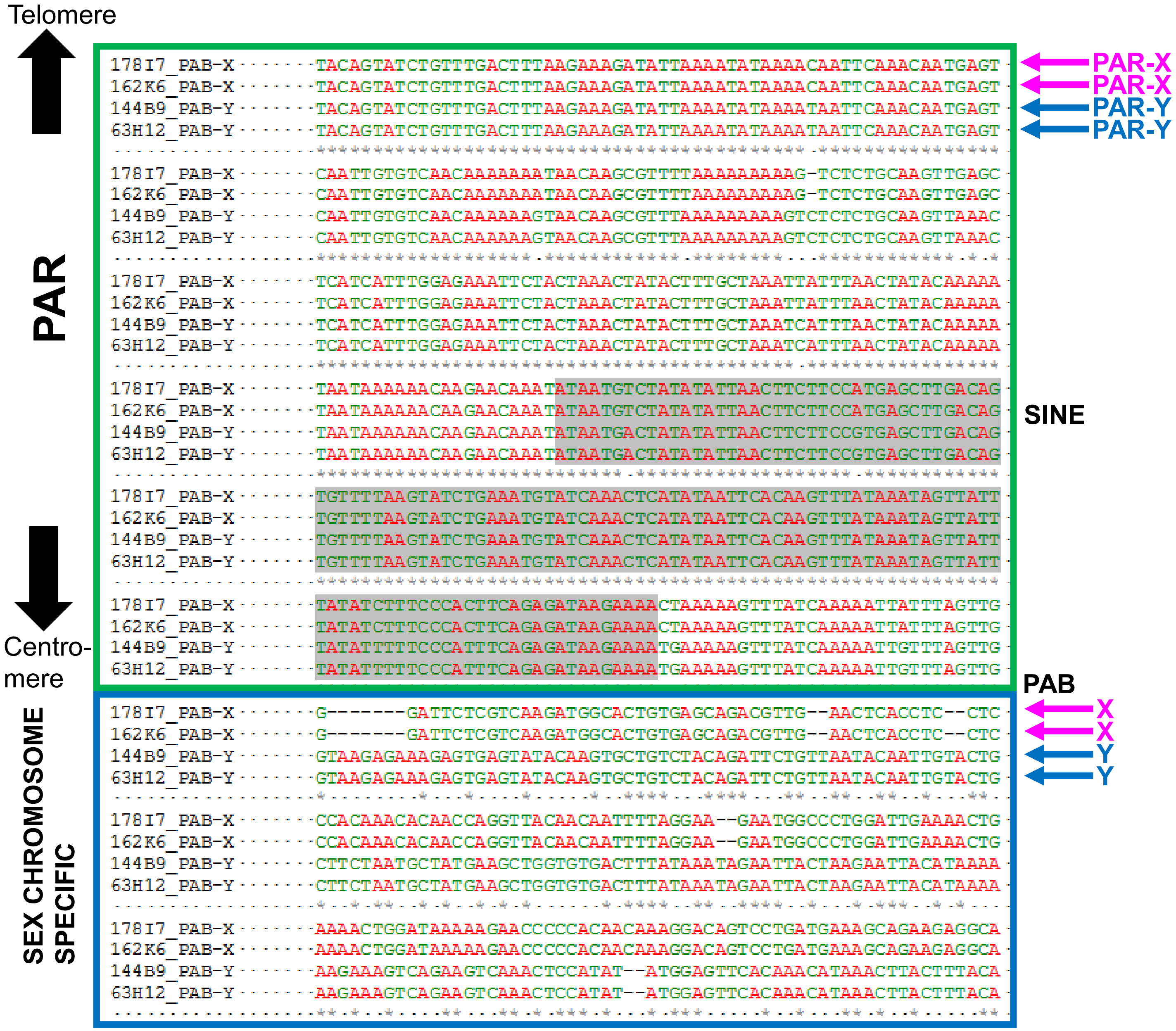

Figure S1. Sequence alignment of four horse BAC clones spanning the PAB: clones 178I7 and 162K6 span PAB in the X chromosome (PAB-X) and clones 144B9 and 63H12 span PAB in the Y chromosome (PAB-Y). A region where all four clones share over 99% sequence identity corresponds to the PAR (green box). A SINE element at the PAB is shaded gray. The lower three lines of alignment (blue box) correspond to sex chromosome specific sequences where sequence similarity between PAB-X and PAB-Y BACs drops dramatically. Note that in both regions sequences of the two PAB-X BACs and the two PAB-Y BACs are 100% identical.

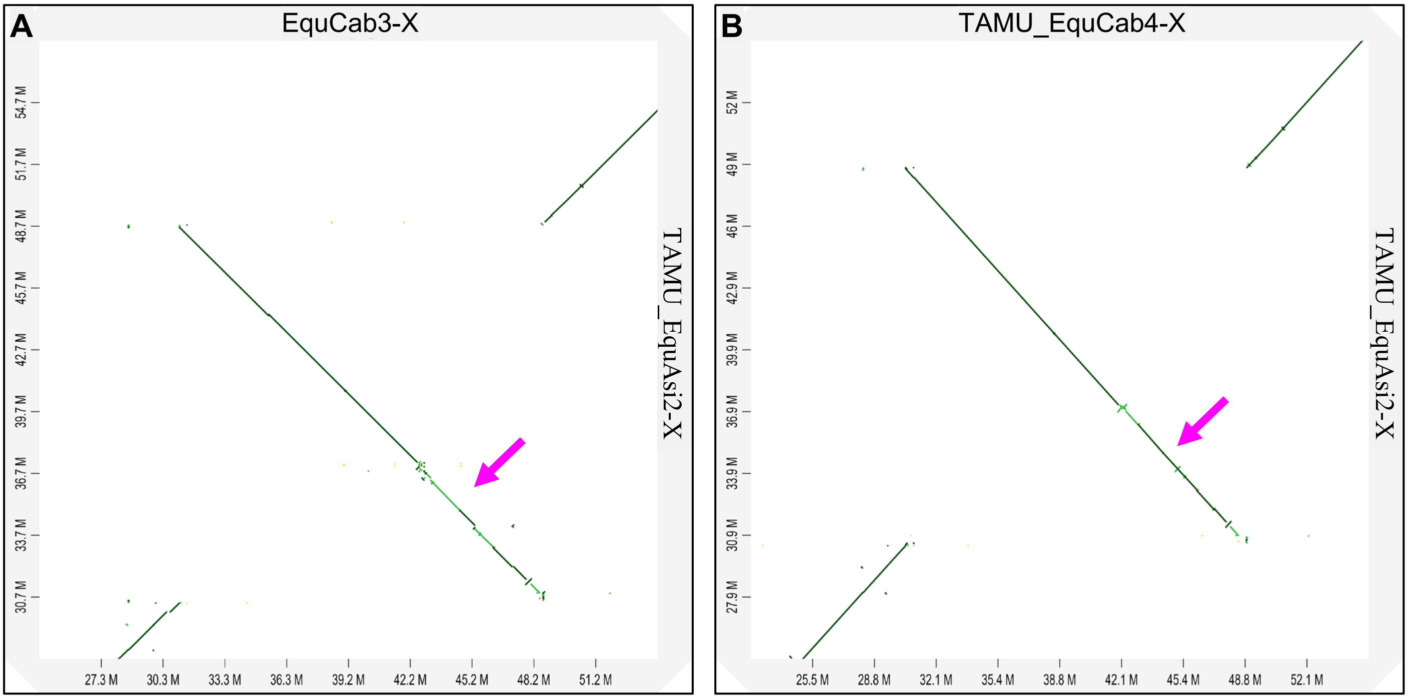

Figure S2. Pericentric inversion in the donkey X chromosome. Alignment dot plots (A) between EquCab3-X and TAMU_EquAsi2-X and (B) TAMU_EquCab4-X and TAMU_EquAsi2-X map the distal inversion breakpoint approximately to 31 Mb and the proximal breakpoint to approximately 49 Mb in the horse X chromosome. Note the reduced horse-donkey alignment accuracy in the proximal portion of the inversion (arrows), likely due to centromeres.

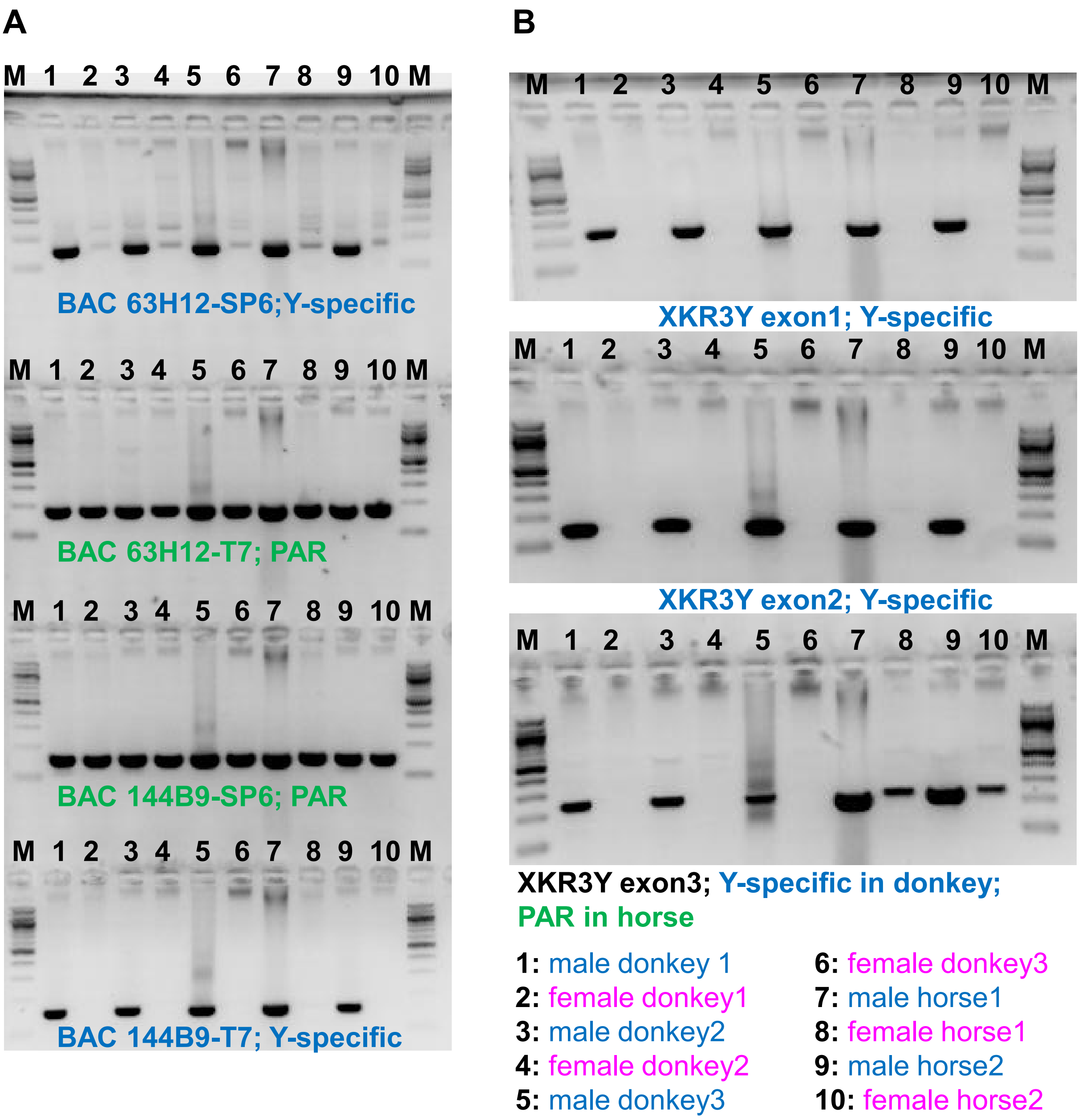

Figure S3. **PCR analysis of the horse and donkey PAB and the *XKR3Y* gene.** (**A**) PCR results on gDNA of male and female donkeys and horses using end sequence primers of the two BAC clones that span PAB in the horse Y chromosome (Raudsepp and Chowdhary, 2008), and (**B**) PCR results on gDNA of male and female donkey and horses using primers specific for the three exons of the horse *XKR3Y* gene.

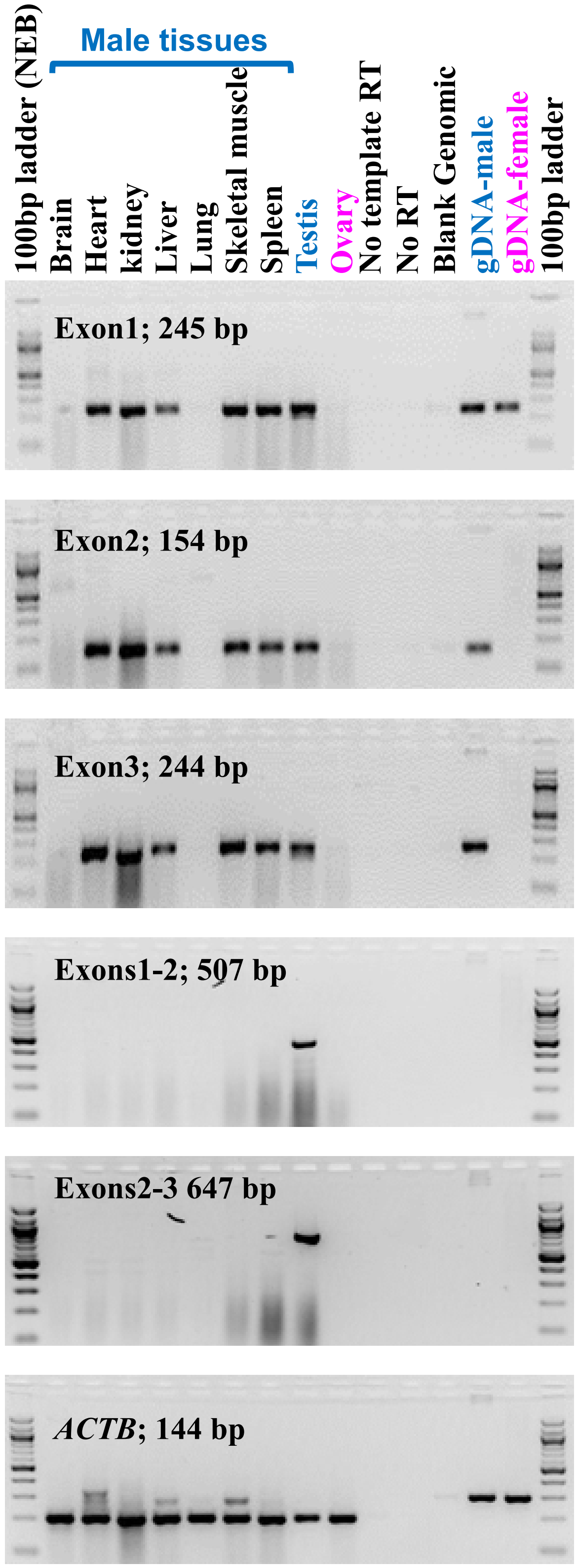

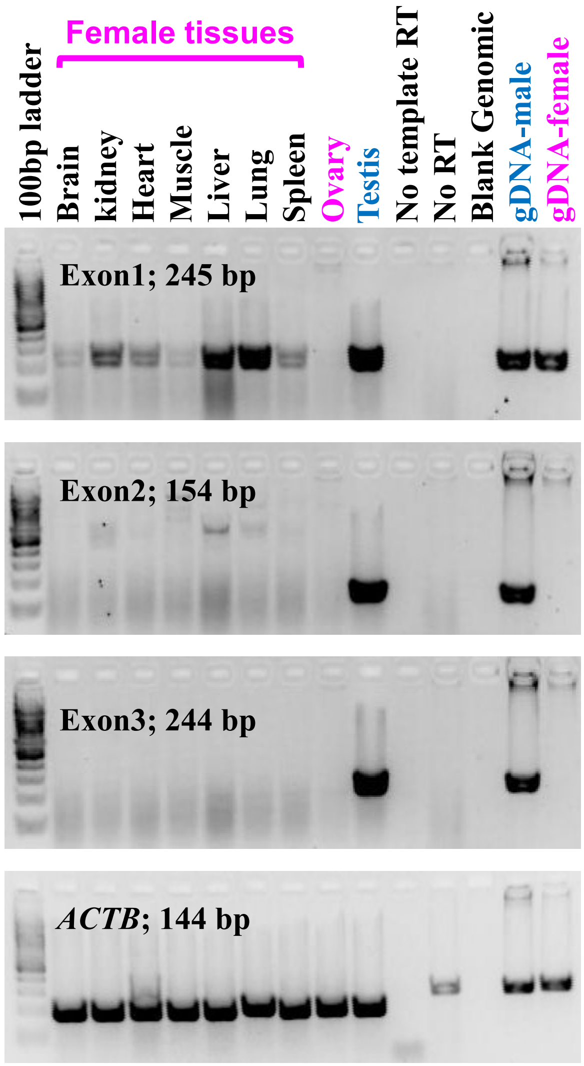

Figure S4. Reverse transcriptase PCR on male (left) and female (right) somatic tissues, testis and ovary using horse *XKR3Y* exonic primers and their combinations showing that exons 1 and 2 are male specific and exon 3 (PAR) expressed in male and female somatic tissues. Isoforms comprised of exons 1 and 2 and exons 2 and 3 are exclusively expressed in testis.

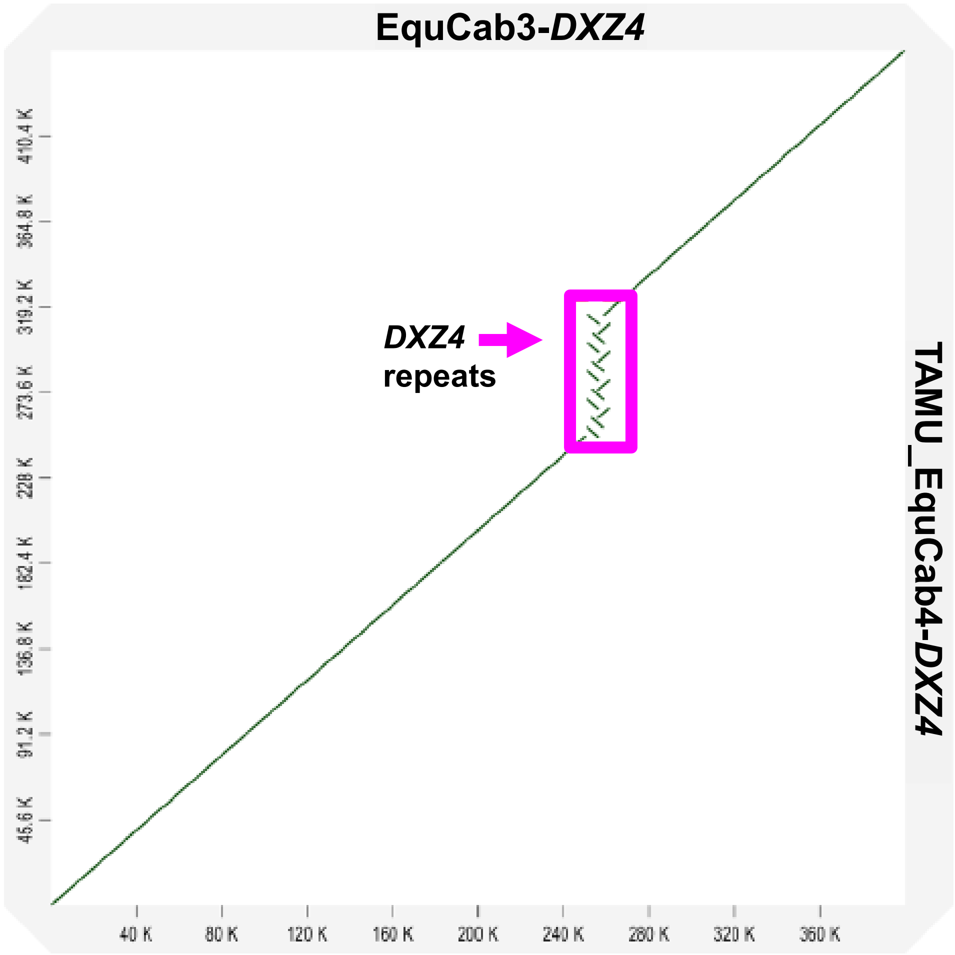

Figure S5. Mashmap alignment of regions surrounding *DXZ4*. EquCab3-*DXZ4* (x-axis) and TAMU_EquCab4-*DXZ4* (y-axis); a collapsed sequences in EquCab3-X are in pink box.

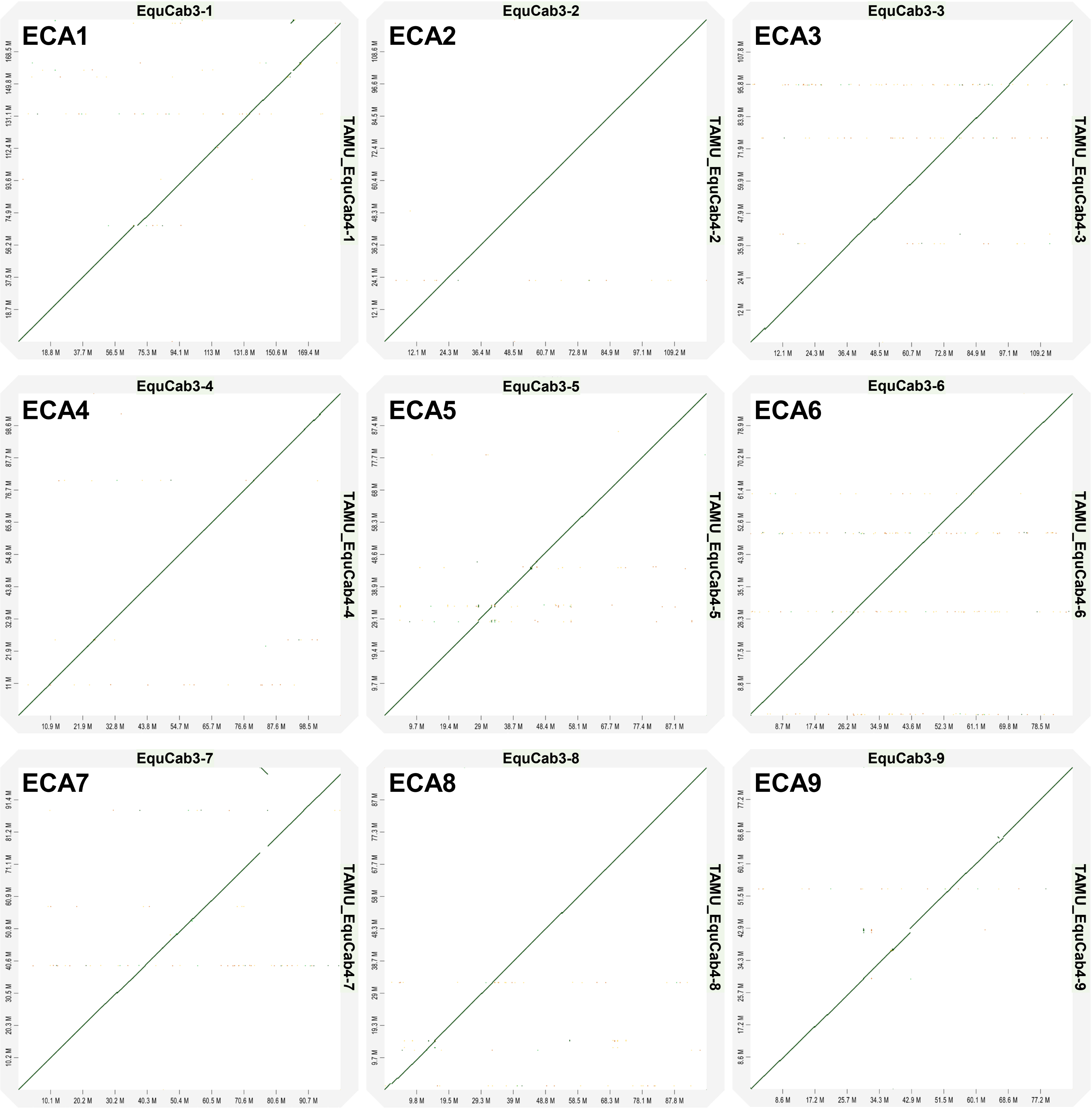

Figure S6. Alignment dot plots of all TAMU_EquCab4 (y-axis) and EquCab3 (x-axis) autosomes; the alignments were done with Minimap2 function of D-Genies.

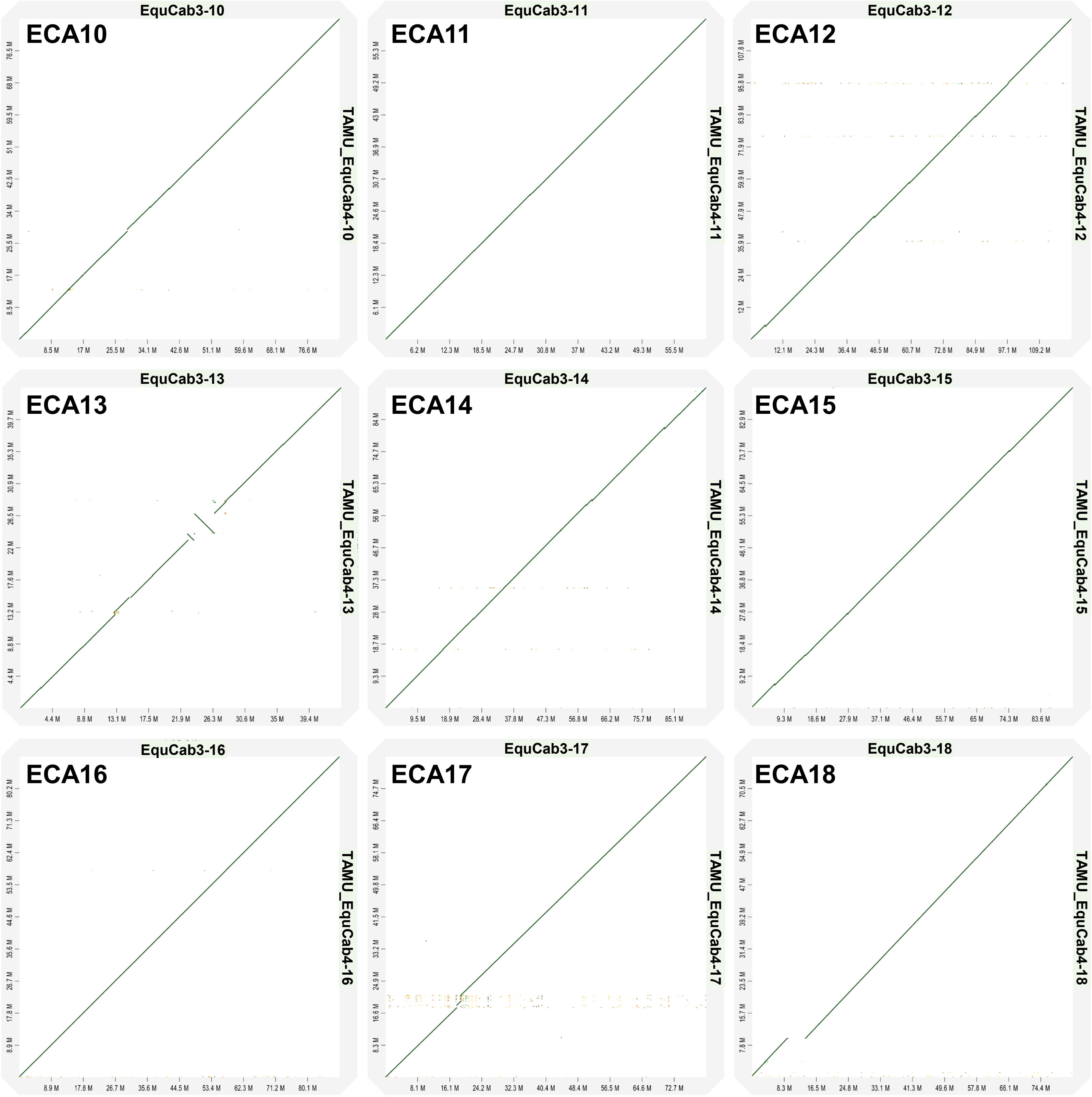

Figure S6. Alignment dot plots of all TAMU_EquCab4 (y-axis) and EquCab3 (x-axis) autosomes; the alignments were done with Minimap2 function of D-Genies.

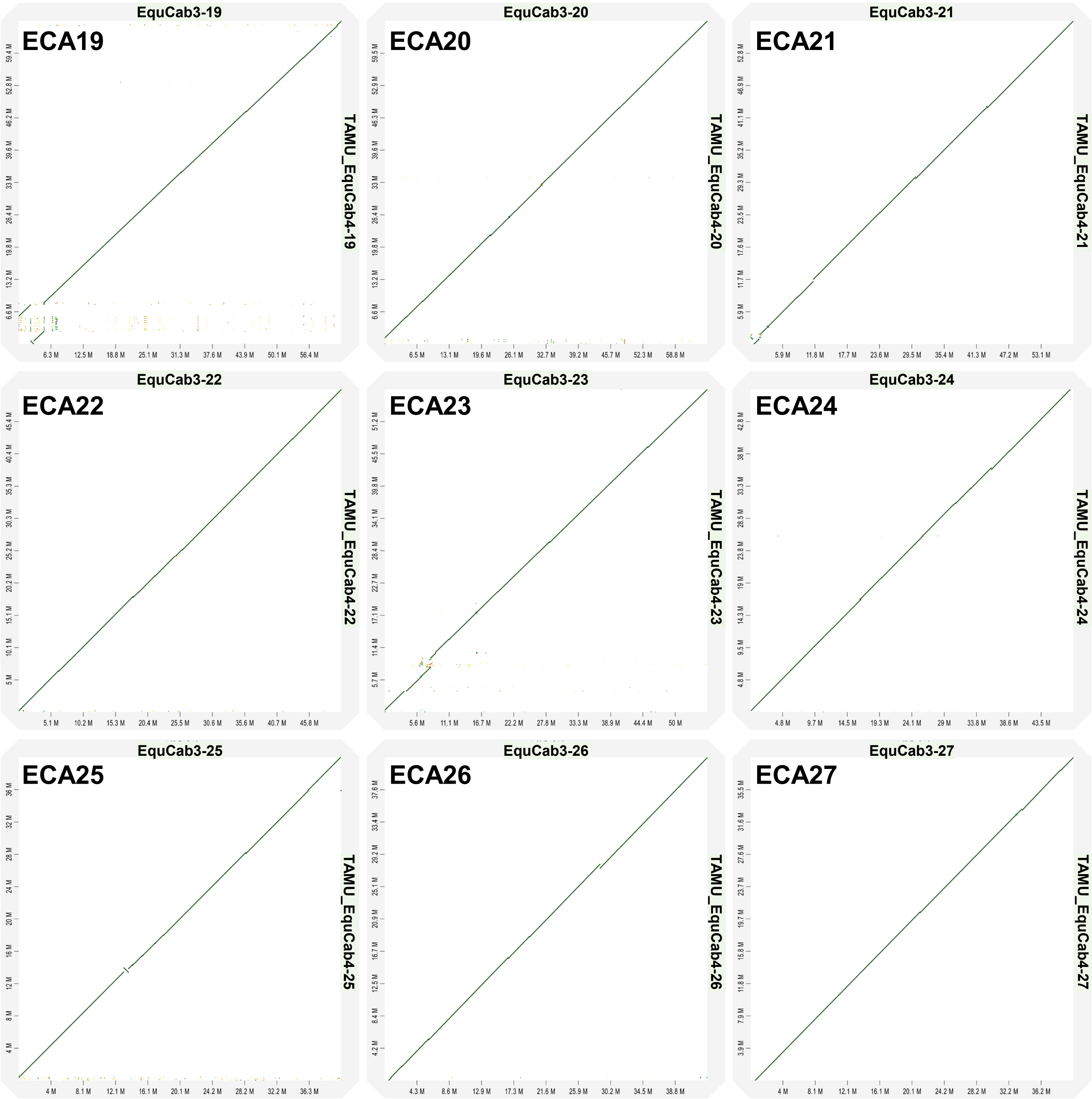

Figure S6. Alignment dot plots of all TAMU_EquCab4 (y-axis) and EquCab3 (x-axis) autosomes; the alignments were done with Minimap2 function of D-Genies.

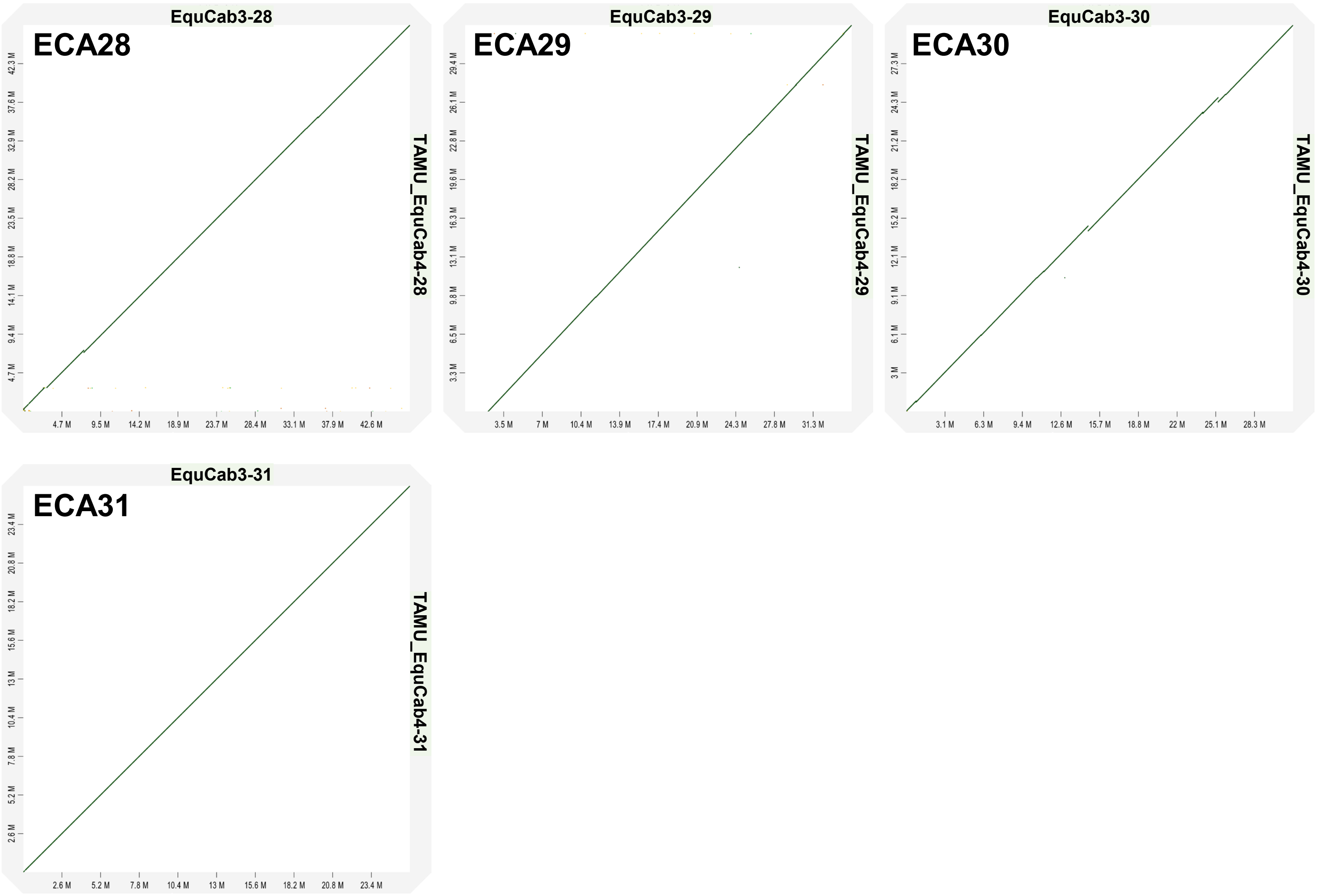

Figure S6. Alignment dot plots of all TAMU_EquCab4 (y-axis) and EquCab3 (x-axis) autosomes; the alignments were done with Minimap2 function of D-Genies.

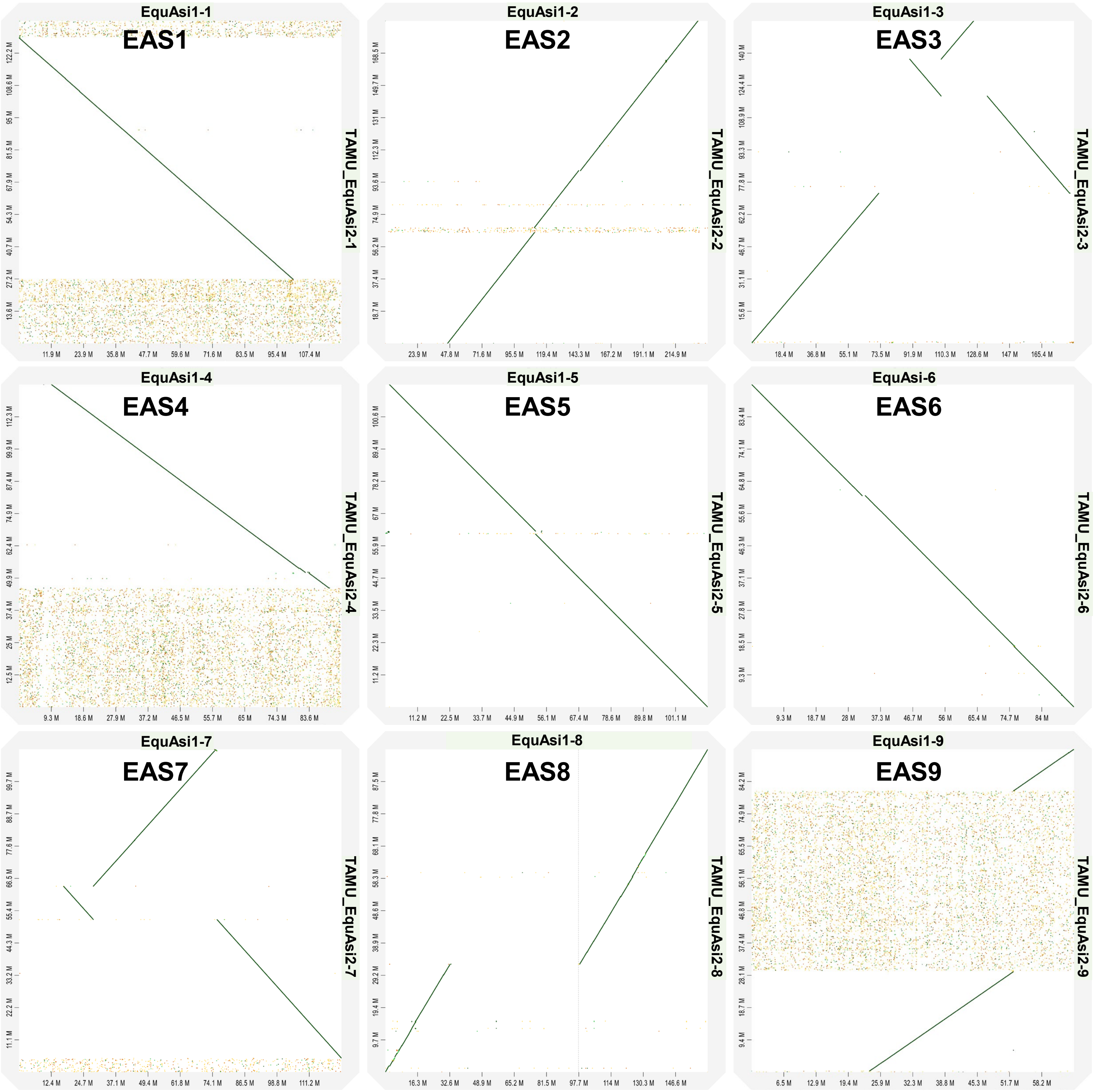

Figure S7. Alignment dot plots of all TAMU_EquAsi2 (y-axis) and EquAsi1 (x-axis) autosomes; the alignments were done with Minimap2 function of D-Genies; detailed comments about assembly problems in EquAsi1 and corrections made in TAMU_EquAsi2 are listed in Table S7.

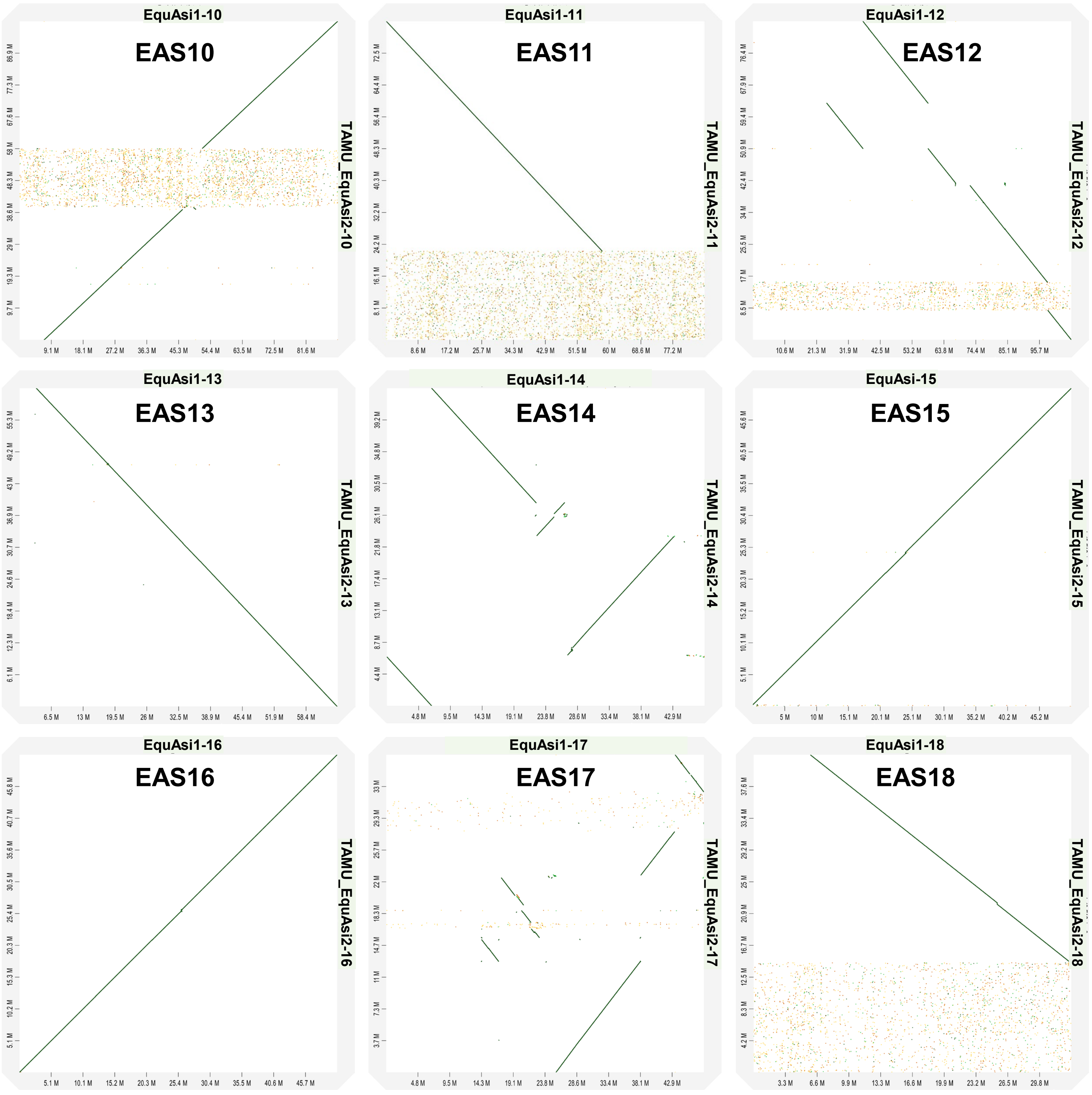

Figure S7. Alignment dot plots of all TAMU_EquAsi2 (y-axis) and EquAsi1 (x-axis) autosomes; the alignments were done with Minimap2 function of D-Genies; detailed comments about assembly problems in EquAsi1 and corrections made in TAMU_EquAsi2 are listed in Table S7.

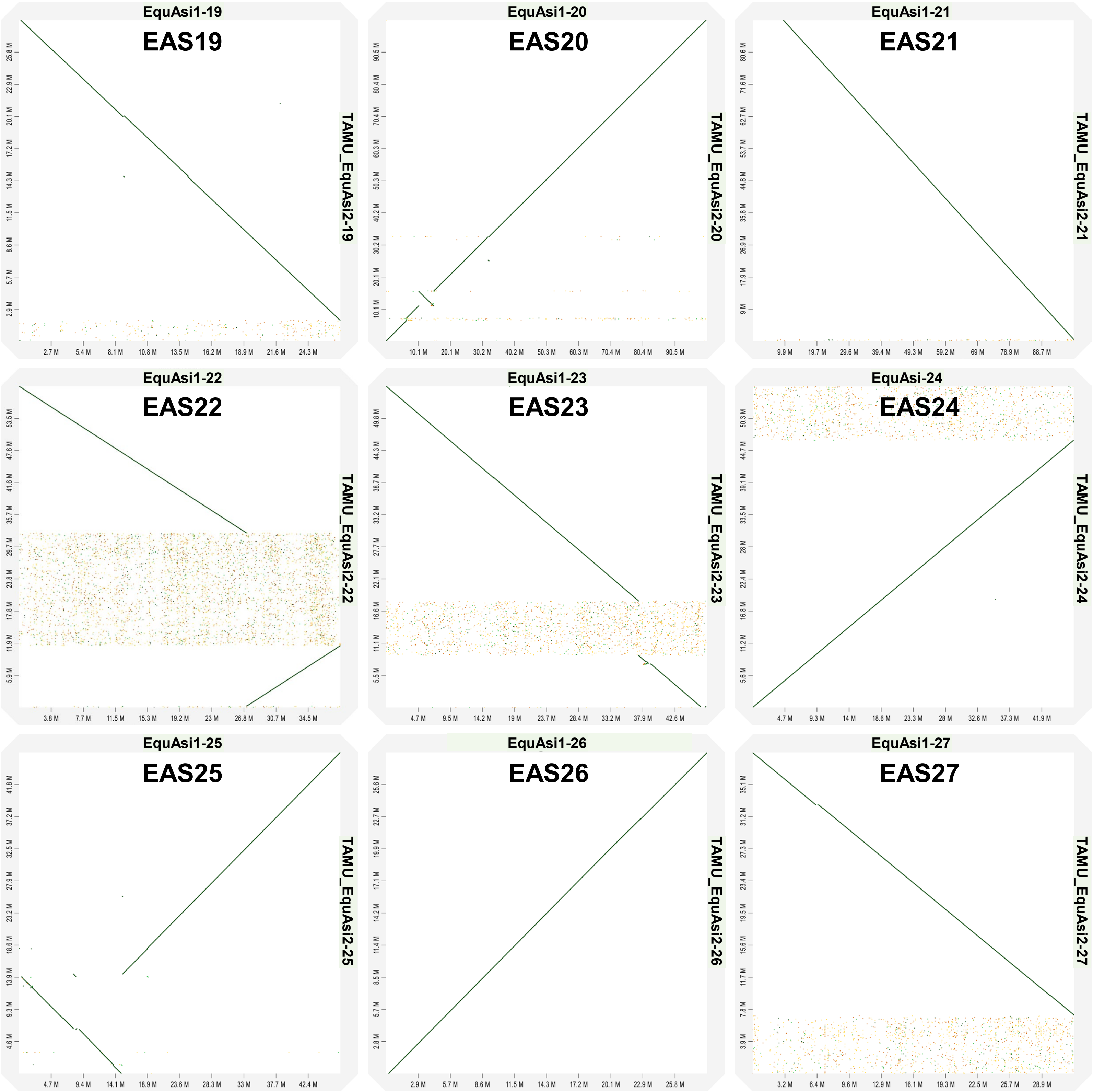

Figure S7. Alignment dot plots of all TAMU_EquAsi2 (y-axis) and EquAsi1 (x-axis) autosomes; the alignments were done with Minimap2 function of D-Genies; detailed comments about assembly problems in EquAsi1 and corrections made in TAMU_EquAsi2 are listed in Table S7.

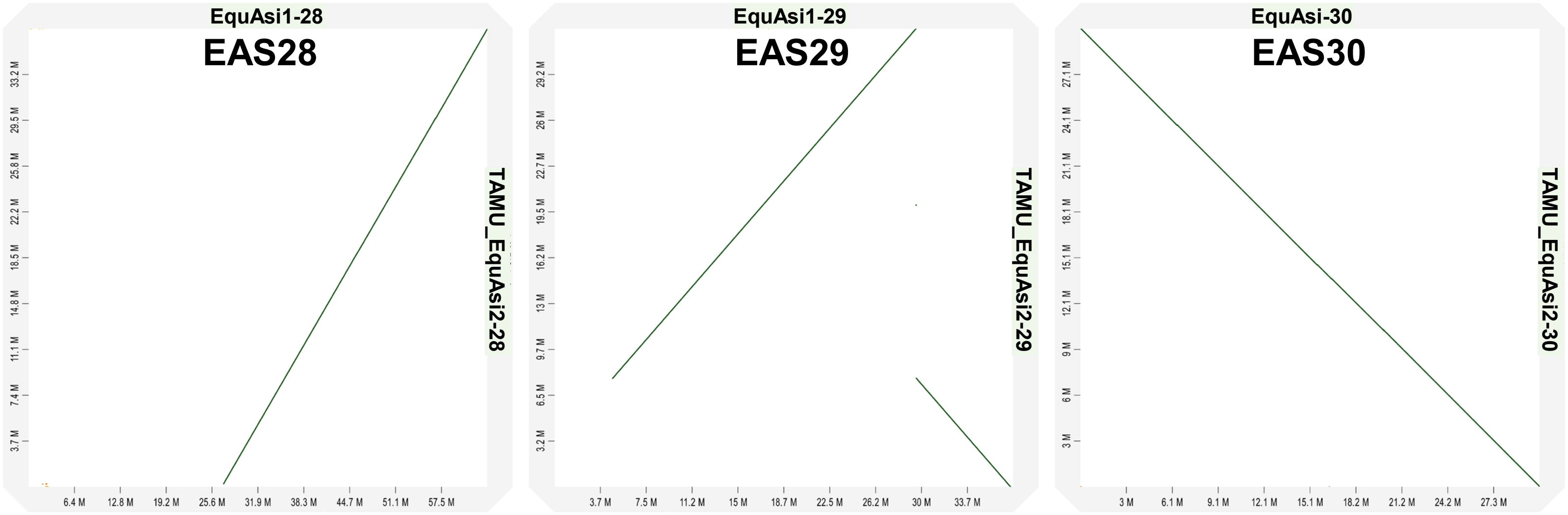

Figure S7. Alignment dot plots of all TAMU_EquAsi2 (y-axis) and EquAsi1 (x-axis) autosomes; the alignments were done with Minimap2 function of D-Genies; detailed comments about assembly problems in EquAsi1 and corrections made in TAMU_EquAsi2 are listed in Table S7.

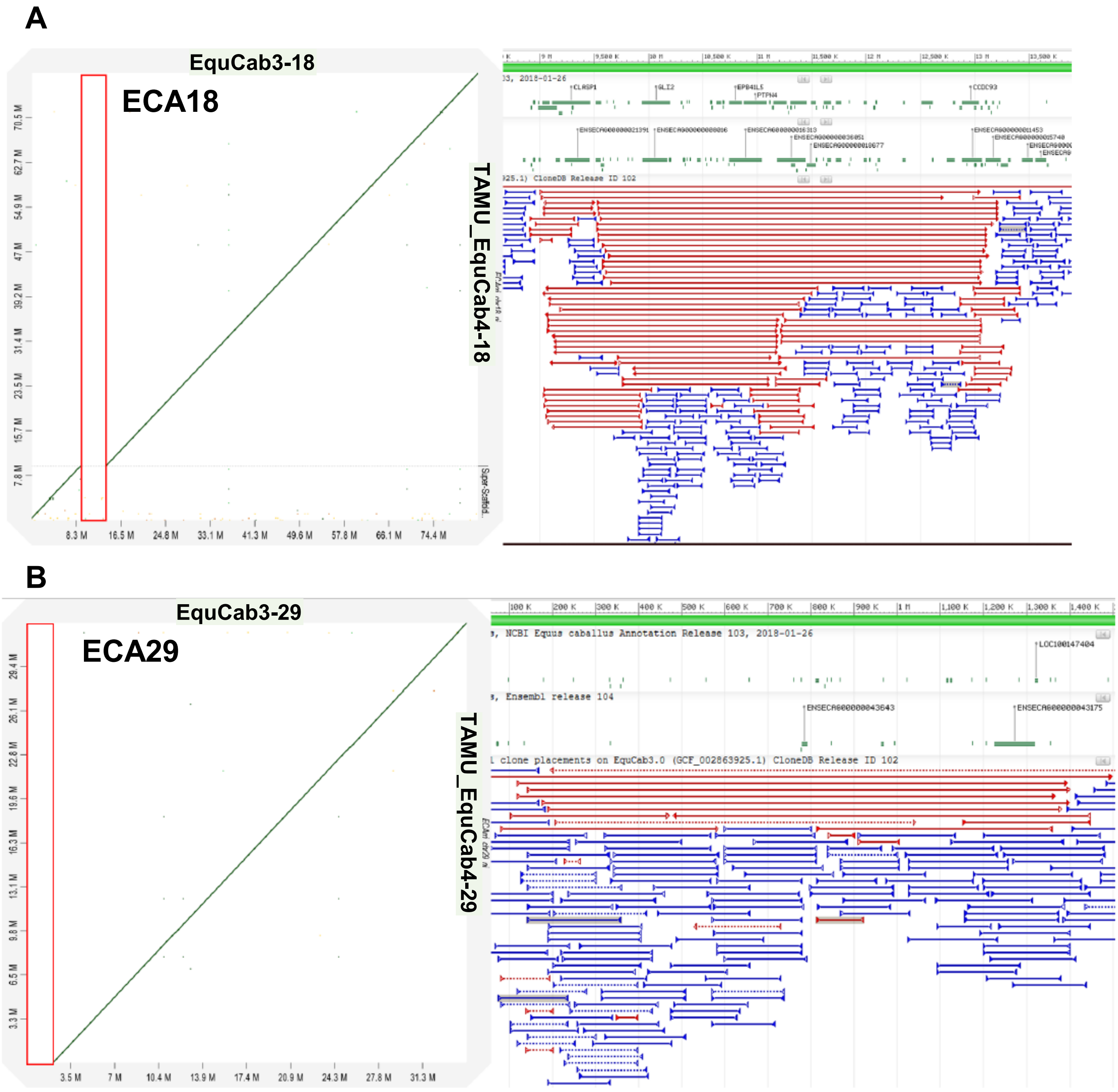

Figure S8. Corrections in horse autosomal assembly for ECA18 (A) and ECA29 (B). (A-left) Alignment of ECAnp4-18 (y-axis) to EquCab3-18 (x-axis). The red box marks the sequences present in EquCAb3-18 but not present in ECAnp4-18. **(A-right)** The missing region is present in EquCab3-18 viewed with the BAC clone tack. The red lines correspond to BACs which ends are discordantly mapped in this sequence indicating it may be assembled incorrectly. **(B-left)** Alignment of ECAnp4-29 (y- axis) to EquCab3-29 (x-axis). The red box marks the sequences present in EquCab3-29 but not present in ECAnp4-29. **(B-right)** The missing region is present in EquCab3-29 viewed with the BAC clone tack. The red lines correspond to BACs which ends are discordantly mapped in this sequence indicating it may be assembled incorrectly.
