# Supplementary material for: Trio-binning of a hinny refines the comparative organization of the horse and donkey X chromosomes and reveals novel species-specific features
Source: Sci Rep. 2023 Nov 17;13:20180. doi: 10.1038/s41598-023-47583-x (PMC10656420; doi:10.1038/s41598-023-47583-x)
Supplement: Supplementary file 2 — Supplementary Tables. [file 41598_2023_47583_MOESM2_ESM.pdf]

Trio-binning of a hinny refines the comparative organization of the horse and donkey X chromosomes and reveals novel species-specific features

Matthew J. Jevit[1], Caitlin Castaneda[1], Nandina Paria[2], Pranab J. Das[3], Donald Miller[4], Douglas F. Antczak[4], Theodore S. Kalbfleisch[5], Brian W. Davis[1]*, and Terje Raudsepp[1]*

[1] School of Veterinary Medicine, Texas A&M University, College Station, TX 77843, USA
[2] Texas Scottish Rite Hospital for Children, Dallas, TX 75219, USA
[3] ICAR-National Research Centre on Pig, Rani, Guwahati, Assam 781131, India
[4] Baker Institute for Animal Health, Cornell University, Ithaca, NY 14853, USA
[5] Maxwell H. Gluck Equine Research Center, University of Kentucky, Lexington, KY 40546, USA

*Corresponding authors:
Terje Raudsepp traudsepp@cvm.tamu.edu
Brian W. Davis bdavis@cvm.tamu.edu

Table S1: Primers for PCR and RT-PCR

Table S2: Sequence of intra-genic TATA box in the *XKR3Y* gene

Table S3: Size, sequence positions and completeness of DXZ4 repeats in horse and donkey X chromosome assemblies; only alignments over 500 bp are included

Table S4: Horse and donkey whole genome assembly metrics and comparative metrics with reference assemblies and human T2T assembly

Table S5: Assembly details for each TAMU_EquCab4 chromosome; * - satellite-free centromere

Table S6: Assembly details for each TAMU_EquAsi2 chromosome; * - satellite-free centromere

Table S7: Reliability of donkey chromosome assemblies in the current EquAsi1 reference and corrections made in the new assembly TAMU_EquAsi2

Table S1: Primers for PCR and RT-PCR

Species	Gene/sequence	Exon/region	Forward 5'-3'	Reverse 5'-3'	gDNA product, bp	cDNA product, bp
Horse	*XKR3*	exon1 F/R	AGTGGTCCTTCGCCACATAC	TGTGTTACTTTTGGGGCACA	245	N/A
Horse	*XKR3*	exon2 F/R	ACGACGCTGGAGACAGAGAT	CTCACAATAAGAGAATGGCCTT	154	N/A
Horse	*XKR3*	exon3 F/R	ATGGGGCTATTCACTGCAAC	TGGAGAAGTGGAGCTCACCT	244	N/A
Horse	*XKR3*	exon1 F/exon2 R	AGTGGTCCTTCGCCACATAC	CTCACAATAAGAGAATGGCCTT	9563	507
Horse	*XKR3*	exon2 F/exon3 R	ACGACGCTGGAGACAGAGAT	TGGAGAAGTGGAGCTCACCT	3625	647
Horse	*ACTB*	exons2 F-exon3 R	CGTCTTCCCCTCCATCGT	AGGTGTGGTGCCAGATCTTC	297	183

Table S2: Sequence of intra-genic TATA box in the *XKR3Y* gene

Origin	Sequence
**TAMU_EquCab4-X**	*XKR3Y* -ACAAACAATAAACACATGCATACATTTACACACACATATATA-PAB
**eMSYv3**	*XKR3Y* -ACAGACAATAAACACATGCATACATTTACACACACATATATA -PAB

Table S3: Size, sequence positions and completeness of DXZ4 repeats in horse and donkey X chromosome assemblies; only alignments over 500 bp are included

Assembly	Size	Full/Partial	Start	End	Region size, bp
TAMU_EquCab4-X	8,049	full	110,543,681	110,551,730	
	8,036	full	110,551,273	110,559,309	
	8,039	full	110,528,541	110,536,580	
	8,017	full	110,536,122	110,544,139	
	8,056	full	110,513,363	110,521,419	
	8,048	full	110,520,951	110,528,999	
	8,073	full	110,498,173	110,506,246	
	8,064	full	110,505,769	110,513,833	
	8,040	full	110,490,593	110,498,633	
	2,614	partial	110,558,853	110,561,467	
	964	partial	110,490,104	110,491,068	71,363
EquCab3-X	8,037	full	95,859,271	95,851,234	
	3,910	partial	95,846,280	95,850,190	
	2,157	partial	95,859,297	95,861,454	
	954	partial	95,845,791	95,846,745	15,663
TAMU_EquAsi2-X	8029	full	96491577	96499605	
	8030	full	96476424	96484436	
	8031	full	96468847	96476860	
	8031	full	96484000	96492013	
	8029	full	96446113	96454124	
	8033	full	96461268	96469283	
	8034	full	96453688	96461704	
	8034	full	96438533	96446549	
	2630	partial	96499169	96501795	
	927	partial	96438045	96438969	63,750
Scaffold 127	8033	full	48684	40661	
	8030	full	41097	33085	
	8032	full	33521	25507	
	8032	full	63841	55827	
	8032	full	78996	70982	
	8031	full	71418	63405	
	8033	full	56263	48248	
	8031	full	25943	17934	
	8044	full	18370	10353	
	8032	full	86562	78560	
	8038	full	10788	2782	
	8077	full	94140	86127	
	4293	partial	97940	93704	
	3238	partial	3217	1	
EquAsi1-X	2626	partial	95519894	95517270	
	2203	partial	95524954	95522809	
	1642	partial	95522779	95521138	
	1548	partial	95521005	95519458	2,915

Table S4: Horse and donkey whole genome assembly metrics and comparative metrics with reference assemblies and human T2T assembly

	**Horse assembly metrics**			**Donkey assembly metrics**		
**Assembly parameters**	**Horse-contig**	**Horse-scaffold**	**TAMU_EquCab4**	**Donkey-contig**	**Donkey-scaffold**	**TAMU_EquAsi2**
Total size; bp	2,527,288,541	2,576,819,369	2,576,761,016	2,485,351,524	2,494,537,995	2,494,488,179
Max size; bp	93,345,358	94,133,619	187,258,966	89,159,166	110,822,766	187,186,459
Average size; bp	1,438,411.20	1,639,198.10	1,667,806.50	1,926,629	2,034,696.60	2,083,950.02
N50; bp	41,516,585	50,787,221	87,715,332	36,551,723	55,343,119	96,591,109
L50	23	18	12	24	18	10
total number contigs/scaffolds	1,757	1,572	1,545	1,290	1,226	1,197
percent in chr	na	na	93.7	na	na	95.74

Comparison with reference assemblies and human T2T genome

	**Horse**			**Donkey**		**Human T2T**
**Assembly parameters**	TAMU_EquCab4	EquCab3; Kalbfleisch et al. 2018	EquCab2; Wade et al. 2009	TAMU_EquAsi2	EquAsi1; Wang et al. 2020; NCBI reference	Nurk et al. 2022
Total assembly size; Gb	2.57	2.41	2.47	2.49	2.43	3.1
Scaffold N50; Mb	97	86	46.75	97	98.59	150.6
Number of scaffolds	1,545	4,701	9,687	1,197	50	24

Table S5: Assembly details for each TAMU_EquCab4 chromosome; * - satellite-free centromere

Horse chromosome	Morphology	TAMU_EquCab4-chr size (bp)	Scaffolds	Scaffold size (bp)	Scaffold orientation compared to equCab3
ECA1	bi-armed	187,258,966	100009	67,615,345	reverse complement
			100001	94,133,619	reverse complement
			5014	25,515,722	same as reference
ECA2	bi-armed	120,709,419	100020	48,966,151	same as reference
			10rc	71,748,858	reverse complement
ECA3	bi-armed	119,802,078	2	36,632,186	reverse complement
			3344	83,175,774	reverse complement
ECA4	bi-armed	109,606,422	100044	25,851,497	reverse complement
			100005	83,759,588	reverse complement
ECA5	bi-armed	97,129,447	100034rc	32,879,471	reverse complement
			100047rc	13,466,047	reverse complement
			18	50,787,221	same as reference
ECA6	bi-armed	87,715,332	1707	28,151,903	same as reference
			100036	32,317,009	same as reference
			100042	27,250,168	same as reference
ECA7	bi-armed	101,540,209	4	44,784,295	same as reference
			100050rc	9,137,861	reverse complement
			6688	47,621,978	same as reference
ECA8	bi-armed	96,679,255	100033	32,266,522	same as reference
			12	64,416,876	same as reference
ECA9	bi-armed	85,786,640	3350	29,535,068	same as reference
			100054	7,144,694	same as reference
			100057rc	5,798,754	reverse complement
			6692	43,311,727	same as reference
ECA10	bi-armed	85,037,498	1678rc	28,736,891	reverse complement
			7rc	56,305,060	reverse complement
ECA11*	bi-armed	61,488,403	100013rc	61,490,448	reverse complement
ECA12	bi-armed	35,940,569	100048rc	12,390,221	reverse complement
			100059rc	4,214,333	reverse complement
			100061rc	4,722,109	reverse complement
			1714rc	14,614,853	reverse complement
ECA13	bi-armed	44,084,819	100056rc	6,687,779	reverse complement
			100055rc	6,146,344	reverse complement
			31	31,252,347	same as reference
ECA14	acrocentric	93,341,905	5	93,345,358	same as reference
ECA15	acrocentric	92,113,494	13	92,118,050	same as reference
ECA16	acrocentric	89,112,214	6	89,116,690	same as reference
ECA17	acrocentric	82,996,931	14	82,998,439	reverse complement
ECA18	acrocentric	78,379,037	1719	9,498,411	reverse complement
			3353	68,884,160	same as reference
ECA19	acrocentric	66,012,348	100060	50,66,890	reverse complement
			16	60,947,451	same as reference
ECA20	acrocentric	66,078,254	30	66,080,715	same as reference
ECA21	acrocentric	58,664,975	100014	58,667,790	same as reference
ECA22	acrocentric	50,472,438	100019	50,474,239	reverse complement
ECA23	acrocentric	56,839,219	100015	56,841,170	same as reference
ECA24	acrocentric	47,550,981	100021	47,553,253	same as reference

ECA25	acrocentric	40,050,604	24	40,051,907	same as reference
ECA26	acrocentric	41,779,657	100028	41,782,245	same as reference
ECA27	acrocentric	39,476,460	25	39,477,650	same as reference
ECA28	acrocentric	46,992,779	66	46,994,095	same as reference
ECA29	acrocentric	32,640,167	27	32,641,526	same as reference
ECA30	acrocentric	30,345,216	32	30,347,161	reverse complement
ECA31	acrocentric	26,003,177	38	26,004,456	same as reference
ECAX	bi-armed	143,200,399	3351	48,660,579	reverse complement
			100032	33,989,383	reverse complement
			100051	9,165,336	n/a
			100053	6,738,259	reverse complement
			100026	44,642,821	same as reference

Table S6: Assembly details for each TAMU_EquAsi2 chromosome; * - satellite-free centromere

Donkey chromosome	Morphology	TAMU_EquAsi2-chr size (bp)	Scaffolds	Scaffold size (bp)	Scaffold orientation compared to EquAsi1
EAS1	bi-armed	135782096	100047	17221140	reverse complement
			77	94269107	reverse complement
			1269	24296234	reverse complement
EAS2	bi-armed	187186459	100009	67099126	reverse complement
			100081	2310666	same as reference
			100056	10969747	same as reference
			100004	83272543	reverse complement
			1358	23538825	same as reference
EAS3	bi-armed	155556531	6	72450531	same as reference
			5	83111595	same as reference
EAS4	bi-armed*	124818238	100021	46098735	reverse complement
			100063	5845492	same as reference
			100007	72877409	reverse complement
EAS5	bi-armed*	111746457	100018	51870556	reverse complement
			33	59879178	same as reference
EAS6	bi-armed	92633112	100362	17583652	same as reference
			100006	75051478	same as reference
EAS7	bi-armed*	110819238	24	110822766	reverse complement
EAS8	bi-armed*	97220254	39	58905453	reverse complement
			63	38318581	same as reference
EAS9	bi-armed*	93581102	22	93583954	reverse complement
EAS10	bi-armed*	96591109	48	40124171	same as reference
			100014	56469854	same as reference
EAS11	bi-armed*	80520413	100048	18545676	reverse complement
			52	61976558	reverse complement
EAS12	bi-armed*	84889928	46	41424226	reverse complement
			45	43467955	same as reference
EAS13	bi-armed*	61460081	100011	61461053	same as reference
EAS14	bi-armed*	43511467	118	6954472	same as reference
			95	36558629	same as reference
EAS15	bi-armed	50658244	100042	24504428	same as reference
			100039	26154839	reverse complement
EAS16	bi-armed*	50834005	75	26032043	reverse complement
			100041	24803487	same as reference
EAS17	bi-armed	36667427	100077	2692471	reverse complement
			105	13854889	reverse complement
			100374	6113503	reverse complement
			100052	14006561	same as reference
EAS18	bi-armed*	41737010	100357	22120920	same as reference
			100046	19617005	same as reference
EAS19	bi-armed*	28681659	100036	28682488	same as reference
EAS20	acrocentric	100527978	121	6377974	same as reference
			100058	9322397	reverse complement
			100017	54714108	same as reference
			100034	30116723	reverse complement
EAS21	acrocentric	89541037	100002	89544404	reverse complement

EAS22	acrocentric	59477287	100032	32341968	reverse complement
			100038	27137302	reverse complement
EAS23	acrocentric	55342096	100016	55343119	reverse complement
EAS24	acrocentric	55901673	29	55903306	reverse complement
EAS25	acrocentric	46468028	100053	13102171	reverse complement
			100028	33366812	same as reference
EAS26	acrocentric	28419801	2653	28421023	reverse complement
EAS27	acrocentric*	38956988	1263	38958195	same as reference
EAS28	acrocentric	36925678	53	36926584	same as reference
EAS29	acrocentric	32457614	100031	32457968	reverse complement
EAS30	acrocentric*	30134926	100033	30136014	reverse complement
EASX	bi-armed*	129101567	26	30682545	reverse complement
			100020	47977471	reverse complement
			124	50442963	same as reference

Table S7: Reliability of donkey chromosome assemblies in the current EquAsi1 reference and corrections made in the new assembly TAMU_EquAsi2

Donkey (EAS) chromosome	Horse (ECA) chromosome(s) by Zoo-FISH (Yang et al. 2004)	EquAsi1 reliability	Rationale to evaluate the reliability of donkey chromosomes in EquAsi1	Corrections made	Zoo-FISH (Yang et al. 2004) support
EAS1	ECA31/ECA4	questionable	~15 Mb of EAS1 does not align to either ECA31 and ECA4. This portion aligns to ECA21 and corresponds to EAS10. First ~1 5 Mb of ECA31 not found in EAS1 indicating that it is incomplete	sequence corresponding to scaf100014 removed from the end of EquiAsi1-1. Sequence corresponding to scaf1269 and 100047 were added to TAMU_EquAsi2-1	scaf100014 aligns to EquCab3-21 and based Zoo-FISH, this sequence was included in TAMU_EquAsi2-10; scaf1269 and 100047 align to EquCab3-31 and 4, respectively, agree with Zoo-FISH and were included into TAMU_EquAsi2-1
EAS2	ECA1	questionable	~47 Mb of EAS2 did not align to ECA1. This portion of EAS2 aligned to ECA28 which corresponded to EAS4	sequence corresponding to scaf100021 was removed from the end of EquAsi1-2	scaf100021 corresponds to EquCab3-28 and based on Zoo-FISH, was included in TAMU_EquAsi2-4
EAS3	ECA2q/ECA3q	rearranged	EAS3 aligns to both ECA2q and ECA3q. There are rearrangements in the portion that aligns with ECA3q	nothing added or removed, discordance was observed between EquAsi1-3 and TAMU_EquAsi2 scaf5	no discordance between scaf5 and EquCab3-3
EAS4	ECA28/ECA18	questionable	EAS4 only includs sequences aligning to ECA18, which correspond to EAS4q, EAS4p corresponds to ECA28 and these sequences are missing from EAS4	48 Mb (scaf 100021) was added to TAMU_EquAsi2-4. These sequences were erroneously assigned to EquiAsi1-2	Scaf100021 aligned to EquCab3-28 and its inclusion to TAMU_EquAsi2-4 agrees with Zoo-FISH
EAS5	ECA19/ECA2p	consistent	aligns to both ECA19 and ECA2p	orientation reversed	EquCab3 was used to get TAMU_EquAsi2-5 in correct orientation
EAS6	ECA15	consistent	aligns to ECA15	orientation reversed	EquCab3-15 used to get TAMU_EquAsi2-6 in correct orientation
EAS7	ECA8q/ECA24	questionable	rearranged and ~35 Mb does not align to ECA24 or ECA8	discordance between scaf24 and EquAsi1-7 observed	no discordance observed between scaf24 and EquCab3-8 or EquCab3-24
EAS8	ECA20/ECA8p	questionable	rearranged and ~20 Mb map to ECA6 which corresponds to EAS22	scaf100032 aligned to EquAsi1-8 but was not included TAMU_EquAsi2-8	scaf100032 maps to EquCab3-6 and based on Zoo-FISH, was included in TAMU_EquAsi2-22
EAS9	ECA14	questionable	incomplete and first 20-25 Mb align to ECA17 which corresponds to EAS11	scaf100048 aligned to EquAsi1-9 but was not included in TAMU_EquAsi2-9. Remaining of EquAsi1-9 was incomplete. An additional 50 Mb was included as a part of scaf22 which had not previously been included EquAsi1-9	scaf100048 aligns to EquCab3-11 and based on Zoo-FISH, was included in TAMU_EquAsi2-11. Scaffold 22 aligns completerly with EquCab3-14 and based on Zoo-FISH, was included in TAMU_EquAsi2-9
EAS10	ECA25/ECA21	consistent	EAS10 aligns to both ECA21 and ECA25	~20 Mb corresponding to scaf100014 was added to TAMU_EquAsi2-10; this sequence was missing from EquAsi1-10	scaf100014 aligns completely to EquCab3-25 and paritally to EquAsi1-10, scaf48 aligns completely to ECA25, and based on Zoo-FISH, were included in TAMU_EquAsi2-10
EAS11	ECA17	questionable	~25 Mb aligns to ECA14 which corresponds to EAS9	a region of EquAsi1-11 corresponding to scaf22, was not included in TAMU_EquAsi2-11. Scaf100048 was included in TAMU_EquAsi2-11; this sequences is missing from EquAsi1-11	scaf 22 aligned to EquCab3-14 and based on Zoo-FISH, was incorporated in TAMU_EquAsi2 -9. Scaf100048 aligned to EquCab3-17 and based on Zoo-FISH, was included in TAMU_EquAsi2 -11
EAS12	ECA9	rearranged	~20-25 Mb align to ECA14 which corresponds to EAS9	first ~25 Mb of EquAsi1-12 correspond to scaf22 and was not included in TAMU_EquAsi2-12. Additionally, ~8 Mb of scaf 46 did not map to EquAsi1-12	scaf22 aligns to EquCab3-14 and based onZoo-FISH, was incorporated in TAMU_EquAsi2 -9. Scaf46 aligns to both EquCab3-9 and EquAsi1-12 but a portion in the middle was missing in TAMU_EquAsi2 -12
EAS13	ECA11	consistent	EAS13 aligns to ECA11	orientation reversed; first ~3 Mb of EquAsi1-13 not found in TAMU_EquAsi2 or TAMU_EquCab4	used ECA11 to get TAMU_EquAsi2-13 in correct orientation
EAS14	ECA13	rearranged	rearranged when aligned to ECA13. First ~5Mb did not align to any horse chromosome	A portion of EquiAsi-14 corresponding to scaf 100036 was not included in our TAMU_EquAsi2-14	scaf100036 aligns to EquCab3-6p and based on Zoo-FISH, was incorporated in TAMU_EquAsi2-19
EAS15	ECA22	consistent	ECA22 and EAS15 are orientated correctly and align completely	no significant change	EquAsi1-15 was in agreement with EquCab3-22
EAS16	ECA5q	consistent	EAS16 and ECA5q are orientated correctly and align completely	no significant change	EquAsi1-16 was in agreement with EquCab3-5q
EAS17	ECA12	questionable	The alignment is highly rearranged	TAMU_EquAsi2-17 shows intra-scaffold rearangements when aligned to EquAsi1-17. **Note: EquCab3-12 and TAMU_EquCab4-12 are the most fragmented and questionable assemblies among horse autosomes**	Less intra-scaffold discordance when compared to EquCab3-12 and TAMU_EquAsi2-12.
EAS18	ECA26	questionable	15 Mb of EAS18 does not align to ECA26 and the first 5-8 Mb align to ECA27 which corresponds to EAS27	~6 Mb of EquAsi1-18 corresponding to scaf1263 was not included in TAMU_EquAsi2-18; ~13-14 Mb corresponding to a portion of scaf100357 was added to TAMU_EquAsi2-18 and was missing from EquAsi1-18	scaf 1263 aligns to EquCab3-27 and based on Zo-FISH, was incorporated in TAMU_EquAsi2-27. Scaf100357 aligned completely to EquCab3-26 and based on Zoo-FISH, was included in TAMU_EquAsi2-18

EAS19	ECA6p	consistent	aligns to ECA6p	orientation reversed; ~ 2 Mb of scaf100036 was missing from EquAsi1-19; the rest of scaf100036 was found in EquAsi1-19	scaffold 100036 maps to ECA6p and based on Zoo-FISH, was assigned to TAMU_EquAsi2-19			
EAS20	ECA7	rearranged	EAS20 aligns with ECA7 with a large inversion which is consistent with cytogenetic data (G-band alignment)	no major changes between EquAsi1-20 and TAMU_EquAsi2-20 though there is a small intra-scaffold inversion. Much larger inversion was observed when TAMU_EquAsi2-20 is aligned to EquCAb3-7	Donkey/horse inversion between EAS20 and ECA7 is consistent with cytogenetic and Zoo-FISH data			
EAS21	ECA16	consistent	First ~10 Mb of EAS21 aligns to ECA10 which corresponds to EAS24. The remainder of EAS21 aligns to ECA16 though in opposite orientation.	the initial ~10Mb of EquAsi-21 correpsonds to scaf29. This was not included in our finalized TAMU_EquAsi2 -21	scaf 29 aligns to EquCab3-10 and based on zooFISH data was incorporeated into TAMU_EquAsi2-24			
EAS22	ECA6q	questionable	EAS22 aligns to ECA6q but is rearranged; ~20 Mb of ECA6q does not align to EAS22, indicating EAS22 may be incomplete	~2/3 of scaf100032 was not found in EquAsi1-22 but was included in TAMU_EquAsi2-22	scaf 100032 aligns completely with EquCAb3-6q and based on Zoo-FISH, was incorporated in TAMU_EquAsi2-22			
EAS23	ECA23	consistent	EAS23 aligns to ECA23; ~8 Mb of ECA23 was not found in EAS23, indicating EAS23 may be incomplete	orientation reversed; ~7 Mb portion in the middle of scaf100016 was not found in EquAsi1-23 but was included in TAMU_EquAsi2-23	scaf100016 aligned completely to EquCan3-23. Remaining portion of scaf100016 also aligned to EquAsi1-23; used EquCab3-23 to get TAMU_EquAsi2-23 in correct orientation			
EAS24	ECA10q	consistent	EAS24 aligns with ECA10q; ~10 Mb of ECA10q missing from EAS24, indicating EAS24 may be incomplete.	last ~9 Mb of scaf 29 was not found in EquAsi1-24 but was included in TAMU_EquAsi2-24	scaf 29 aligns completely to EquCab3-10q and based on Zoo-FISH, was incorporated in TAMU_EquAsi2-24			
EAS25	ECA5p	rearranged	EAS25 is rearranged but aligns to ECA5p	orientation reversed	used EquCab3-5p to get TAMU_EquAsi2-25 in correct orientation			
EAS26	ECA10p	consistent	EAS26 aligns with ECA10p	no significant change	EquAsi1-26 was in agreement with EquCab3-26			
EAS27	ECA27	consistent	~8 Mb of ECA27 is not found in EAS27, indicating that EAS27 may be incomplete	a portion of scaf1263 which did not align to EquAsi1-27 was included in TAMU_EquAsi2-27	scaf 1263 aligns to EquCAb3-27 and based on Zoo-FISH was incorporated into TAMU_EquAsi2-27			
EAS28	ECA3p	questionable	first ~25 Mb of EAS28 correspond to ECA31 which corresponds to EAS1. The remining sequence of EAS28 aligns to ECA3p.	the first ~25 Mb of EquAsi1-28 corresponding to scaf100047 was not included in TAMU_EquAsi2-28	scaf100047 aligns to EquCAb3-31 and based on Zoo-FISH,was incorporeated into TAMU_EquAsi2-1			
EAS29	ECA29	rearranged	EAS29 aligns to ECA29 though is rearranged; ~2 Mb of ECA29 not found in EAS29, indicating it may be incomplete	The first ~3 Mb of EquAsi1-29 corresponding to scaf100052 was not included in TAMU_EquAsi2-29	scaf100052 aligns to EquCAb3-12 and based on Zoo-FISH, was incorporeated into TAMU_EquAsi2-17. Scaf 100033 spans EquCab3-29 and based on Zoo-FISH, was incorporated into TAMU_EquAsi2-29			
EAS30	ECA30	consistent	EAS30 aligns to ECA 30	orientation reversed	EquCab3-30 used to get TAMU_EquAsi2-30 in correct orientation			
X	X	rearranged	gross rearrangements and 15 Mb of ECAX is not aligning to EASX; EASX is rearranged and incomplete.	Significant intra-scaffold discordance was observed between TAMU_EquAsi2-X and EquAsi1-X.	Discordance between TAMU_EquAsi2-X and EquCab3-X was not observed.			
Y	Y	mis-assembled	Aligns with TAMU_EquAsi2-X and TAMU_EquCab4-X in similar pattern but the aligned region is too large for the PAR. EquAsi1-Y contains sequences that belong to the X chromosome					
